# Identification of proteins responding to pathogen-infection in the red alga *Pyropia yezoensis* using iTRAQ quantitative proteomics

**DOI:** 10.1186/s12864-018-5229-1

**Published:** 2018-11-27

**Authors:** Sohrab Khan, Yunxiang Mao, Dong Gao, Sadaf Riaz, Zeeshan Niaz, Lei Tang, Sohaib Khan, Dongmei Wang

**Affiliations:** 1 Key Laboratory of Marine Genetics and Breeding (Ocean University of China), Ministry of Education, Qingdao, 266003 China; 2 Laboratory for Marine Biology and Biotechnology, Qingdao National Laboratory for Marine Science and Technology, Qingdao, 266237 China; 3 College of Marine Life Sciences, Ocean University of China, Qingdao, 266003 China

**Keywords:** Quantitative proteomics, iTRAQ, Differentially expressed proteins, *Pyropia yezoensis*, *Pythium porphyrae*, Omics

## Abstract

**Background:**

*Pyropia yezoensis* is an important marine crop which, due to its high protein content, is widely used as a seafood in China. Unfortunately, red rot disease, caused by *Pythium porphyrae,* seriously damages *P. yezoensis* farms every year in China, Japan, and Korea. Proteomic methods are often used to study the interactions between hosts and pathogens. Therefore, an iTRAQ-based proteomic analysis was used to identify pathogen-responsive proteins following the artificial infection of *P. yezoensis* with *P. porphyrae* spores.

**Results:**

A total of 762 differentially expressed proteins were identified, of which 378 were up-regulated and 384 were down-regulated following infection. A large amount of these proteins were involved in disease stress, carbohydrate metabolism, cell signaling, chaperone activity, photosynthesis, and energy metabolism, as annotated in the KEGG database. Overall, the data showed that *P. yezoensis* resists infection by inhibiting photosynthesis, and energy and carbohydrate metabolism pathways, as supported by changes in the expression levels of related proteins. The expression data are available via ProteomeXchange with the identifier PXD009363.

**Conclusions:**

The current data provide an overall summary of the red algae responses to pathogen infection. This study improves our understanding of infection resistance in *P. yezoensis,* and may help in increasing the breeding of *P. porphyrae-*infection tolerant macroalgae.

**Electronic supplementary material:**

The online version of this article (10.1186/s12864-018-5229-1) contains supplementary material, which is available to authorized users.

## Background

*Pyropia yezoensis* is a red alga that is extensively used as a food, a medicine, a fertilizer, and as a source of chemicals. In China, *P. yezoensis* is grown extensively, and is widely consumed as a seafood in China, Japan, and South Korea [1, 2]. Interestingly, it has been noticed that in Asian countries there is a smaller cancer incidence rate compared to North American and European countries, and this is thought to be due to the consumption of seaweeds [3]. Amongst seaweeds, red algae are a rich source of proteins, minerals, and carbohydrates [4].

Seaweeds can be exposed to several biotic stresses, including infection by *Pythium porphyrae*, which causes red rot disease and has a direct effect on commercial seaweed production. Few studies have addressed disease progression *P. yezoensis,* and very few eukaryotic algal pathogens have been isolated for culture in the laboratory [5]. Red rot disease was first described in 1947 by Arasaki et al. After this, it took a long time to isolate and identify the causative factor i.e., the oomycete *P*. *porphyrae* [6, 7]. Even though the physiological and ecological characteristics of *P*. *porphyrae* have been studied and examined intensively, there are very few studies that address the cellular and molecular mechanisms of infection [8–15].

The disease initiation process in microalgae is very similar to that of plants infected with oomycetes, involving the production of zoospores. The presence and distribution of algal species in aquatic regions results in an interaction between biotic and abiotic factors [16–20]. There is a chance for micro- and macro-algae to adopt under normal occurring abiotic factors, which might contribute to the over-production of reactive oxygen species (ROS) [21–29].

Proteomics is a highly useful method that can be used to explore the molecular changes that occur following infection, but before the obvious expansion of the disease [30], so this method is useful in the assessment of toxicity. Proteomics may be more sensitive at detecting harmful effects at an early phase, since organisms can be exposed to low doses of an infectious agent, thereby improving risk assessment [31]. Proteomics methods have previously been used to investigate the host reaction to viral infections, such as dengue virus [32] and Marek’s disease virus [33]. Prior to the introduction of modern proteomics methods, 2-D gel electrophoresis and the shotgun technique were widely used in proteomics research [34, 35].

Proteomic methods have been used to study the interactions between hosts and pathogens [36–39]. Compared to a transcriptomic analysis, proteomics is better able to uncover the action of effector molecules that account for a particular phenotype [40]. Proteomic studies can provide a detailed insight into alterations in proteins that occur following different type of stresses, and is able to identify potential biomarkers [41, 42]. Quantitative proteomics offers a proactive technique that is able to detect and quantify an organism’s proteome. 2-D gel electrophoresis is normally used for protein quantitation before mass spectrometry [43]. Isobaric tags for relative and absolute quantitation (iTRAQ) is an extensively used method for the relative quantification of peptides, allowing for up to eight samples at the same time [44, 45]. This approach is therefore suitable for examining changes in protein expression levels, and is also able to assess the influence of exposure time on an affected organism, since it can also provide relative quantitation [41, 42, 46].

This study was undertaken in an attempt to understand the mechanism of resistance to infection in order to improve the breeding of *P. yezoensis* that are tolerant to macroalgae. Accordingly, we focused on the interaction between *P. yezoensis* and *P. porphyrae.* The iTRAQ method was used to detect variations in protein expression in *P. yezoensis* infected with oomycetes zoospores. To our knowledge, this is the first report providing a catalog of the proteins expressed in response to disease stress in *P. yezoensis*. This study showed that artificial infection resulted in significant changes in the levels of expression of proteins that may have a role in the algal disease defense mechanism. The significantly altered proteins (pathogen responsive proteins) obtained using the iTRAQ-based analysis were involved in photosynthesis and energy metabolism, disease stress related, cellular processes such as carbohydrate metabolism, chaperone activity, and cell signaling.

## Results

### Overview of proteomic data

We extracted total proteins from three healthy and three infected algal thalli for proteomic analysis. A total of 380,141 mass spectrograms were collected by iTRAQ analysis from both the healthy and infected samples. Some 23,937 unique peptides among the 24,448 total peptides were identified (Table 1), and successfully aligned onto 4011 proteins in the *P. yezoensis* UniGene database [47].

**Table 1:** Statistics for the proteins identified by iTRAQ

Total spectra	Spectra (PS)	Peptides	Unique peptides	Protein groups
380,141	57,994	24,448	23,937	4011

### Differentially expressed proteins (DEPs) identified by iTRAQ

**Fig. 1:**
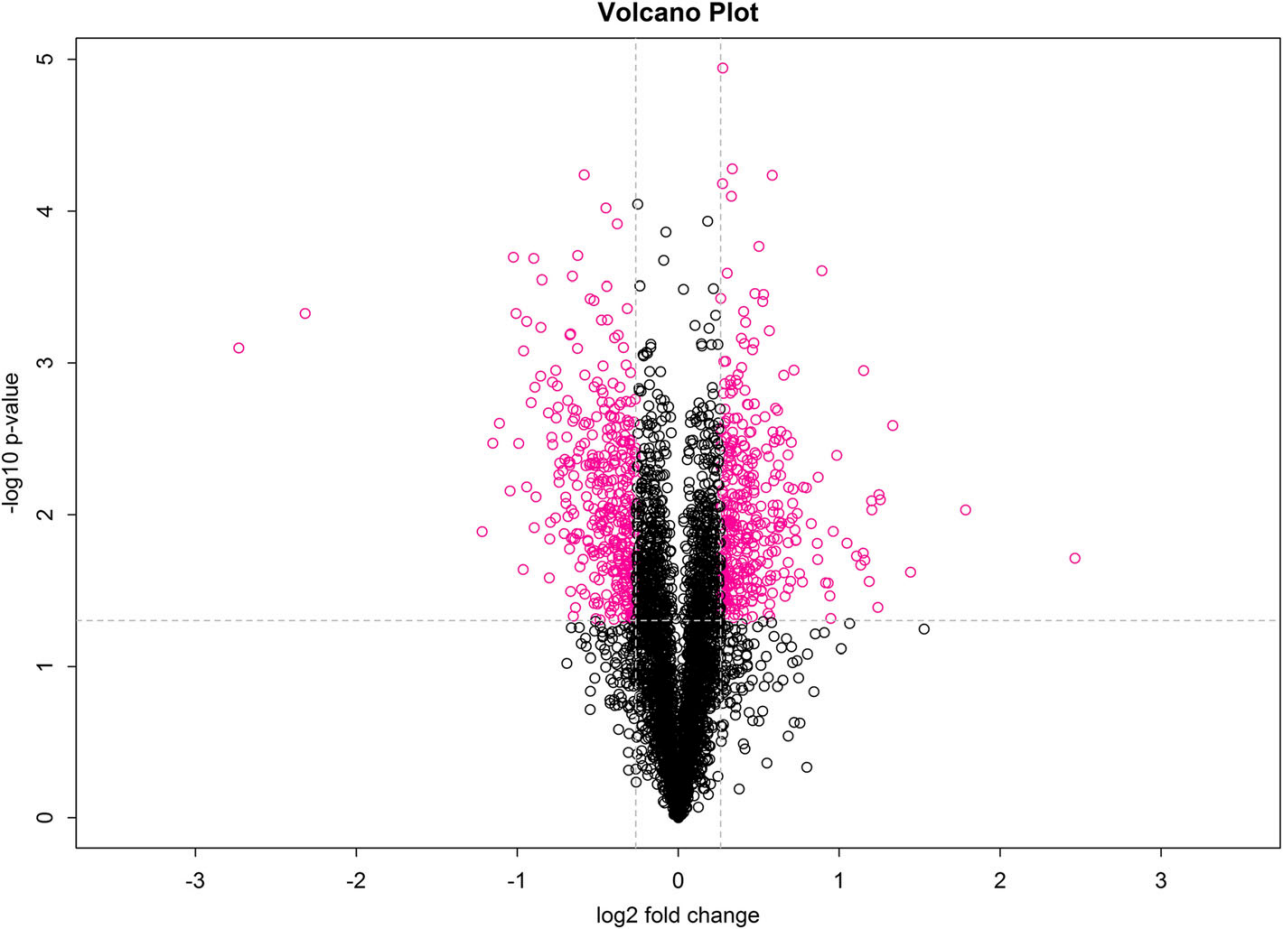
Volcano plot of the identified proteins showing fold changes of > 1.2 or < 0.8 and statistical significant change to a *p*-value of < 0.05 are indicated in red

Based on a *p* value of less than 0.05 and a fold change of > 1.2 or < 0.8 [48, 49], 762 differentially expressed proteins were identified, of which 378 were more highly expressed following infection, and 384 were expressed at lower levels following infection (further details are provided in Additional file 1: Table S1 and Fig. 1).

**Fig. 2:**
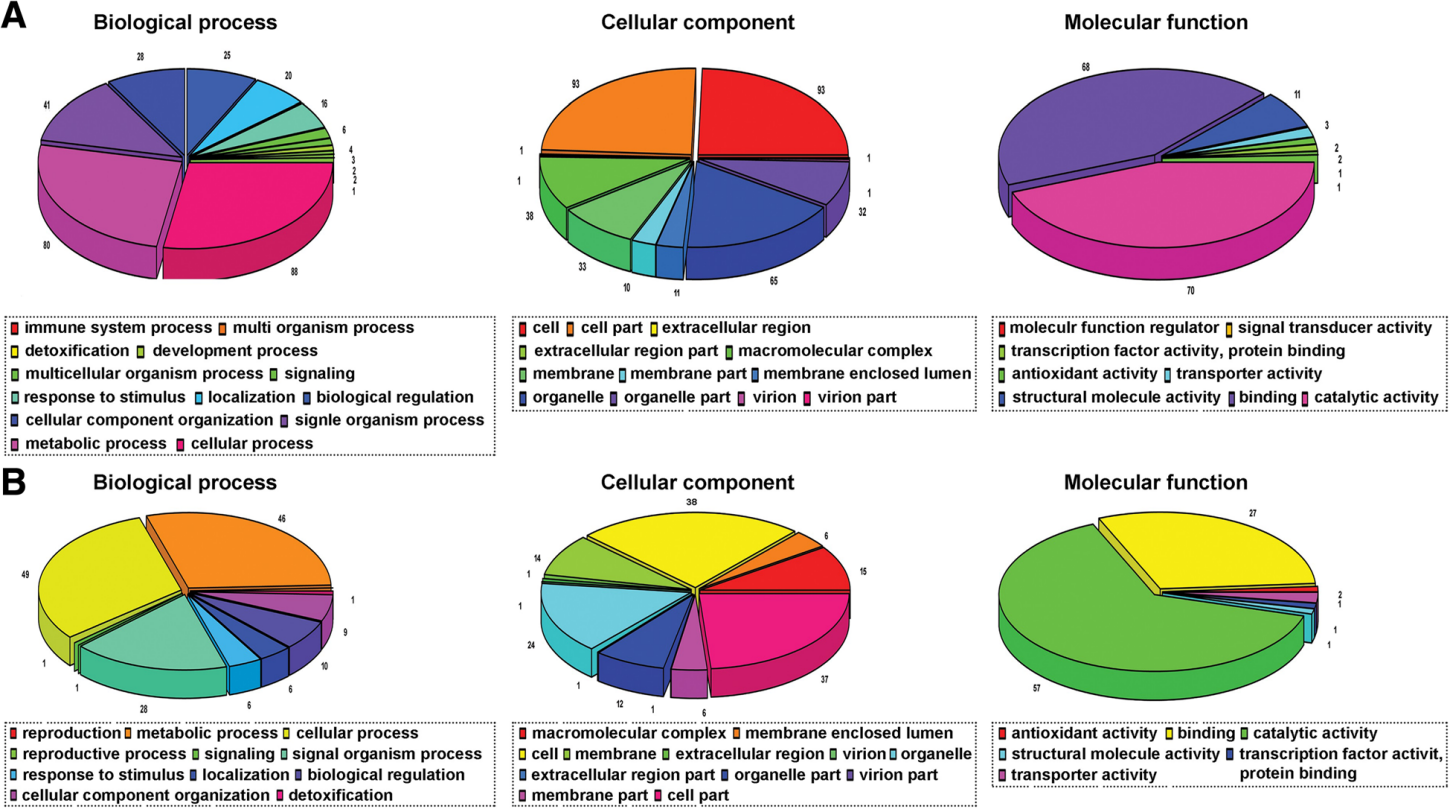
Functional classification of the identified proteins. Proteins were annotated by biological process, cellular component, and molecular function categories. **a** protein categories of the up-regulated differentially annotated proteins (DEPs) **b** protein categories of the down-regulated DEPs

Among the highly DEPs, 151 could be annotated and were found to be enriched in 33 different gene ontology (GO) terms using Blast2GO (Version 3.3.5), as shown in Fig. 2a. These proteins could be characterized into 13 categories according to the GO for biological processes. The main three categories for biological processes that contained a significant number of DEPs were cellular process (88), metabolic process (80), and single organismal process (41). The three main categories for cell components were cell (93), cell part (93), and organelle (65) out of 12 categories; however, the key categories for molecular function were catalytic activity (70), binding (65), and structural molecular activity (11) out of eight categories.

Among the down-regulated DEPs, 103 proteins were annotated by gene ontology, and were found to be enriched in 29 different GO terms (Fig. 2b) using the Blast2GO (Version 3.3.5) bioinformatics software tool. The proteins for biological processes could be placed into 11 categories based on GO terms. The main functional categories containing a large number of DEPs were cellular process (49), metabolic process (46), and single organismal process (28). The three predominant categories for cell components were cell (38), cell part (37), and organelle (24) out of 12 categories, whereas, the three main categories for molecular function were catalytic activity (57), binding (27) and antioxidant activity (2), out of eight categories. These functional categories were important in metabolic and cellular processes, signaling, detoxification, transcription factor activity, protein binding, antioxidant activity, catalytic activity, multi-organism processes, and response to stimulus and developmental and growth processes in *P. yezoensis*.

**Fig. 3:**
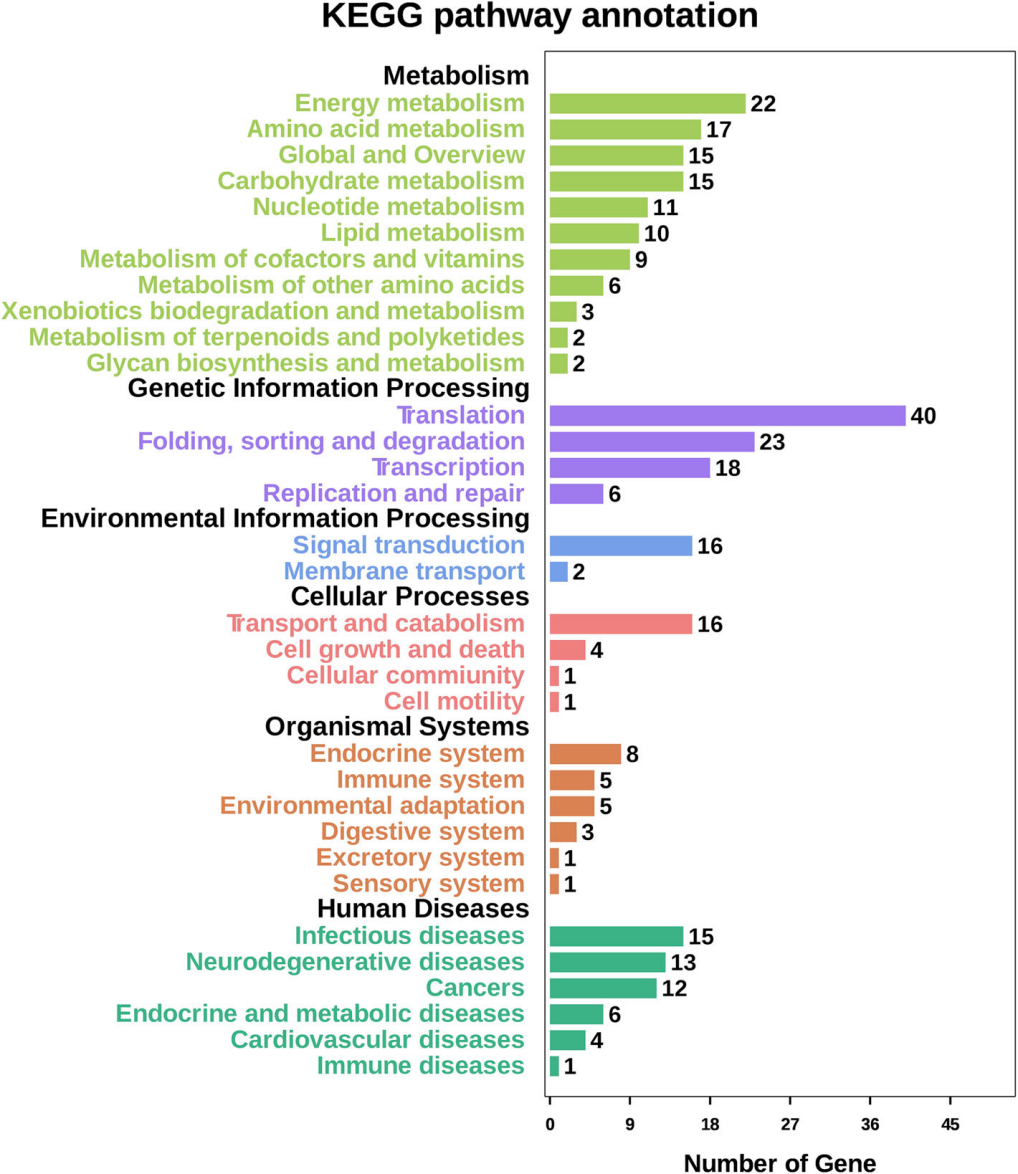
KEGG pathway functional analysis of all the identified DEPs. The X and Y axis represents the number of genes and the different major pathways of metabolic process, genetic, environmental, cellular, organismal, and human diseases pathway information respectively

A KEGG functional analysis indicated that 178 DEPs were involved in 159 pathways that included six major pathways (Fig. 3). The KEGG functional analysis of up-regulated proteins showed that 102 DEPs contributed to 108 different pathways whereas 76 downregulated DEPs were involved in 121 pathways. All the functional categories in these pathways were important, especially infection stress, energy metabolism and photosynthesis, carbohydrate metabolism, and the role of signal transduction pathways in resisting infection.

**Fig. 4:**
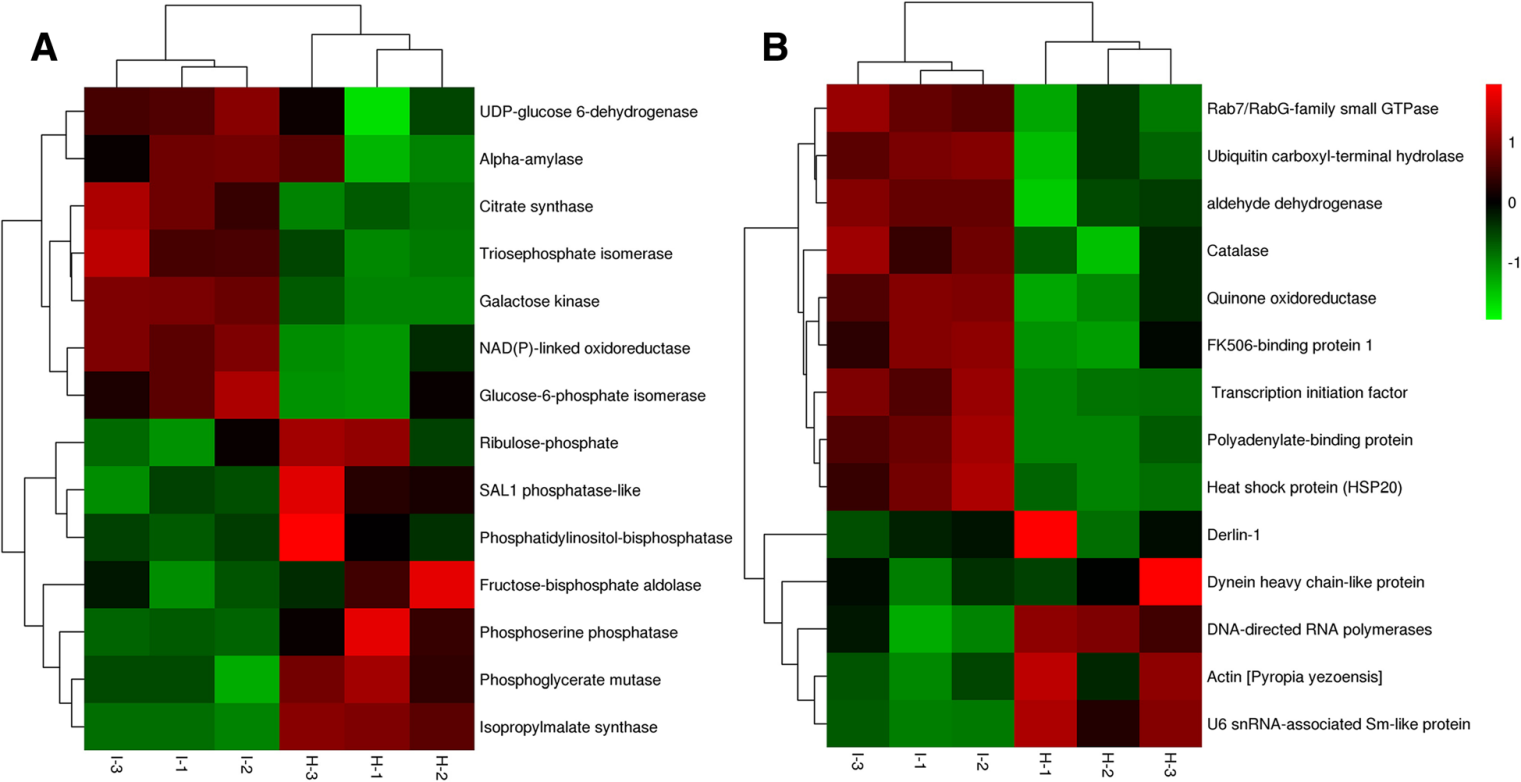
Hierarchical clustering of DEPs under infection stress; **a** Proteins related to carbohydrate metabolism **b** Proteins related to chaperone activity and ROS scavenging. I-1 to I-3 and H-1 to H-3 represent three biological replicates for infected and healthy samples, respectively

With respect to carbohydrate metabolism (Fig. 4a), enzymes such as alpha-amylase, citrate synthase, glucose-6-phosphate isomerase, triosephosphate isomerase, UDP-glucose 6-dehydrogenase, galactose kinase, and NAD(P)-linked oxidoreductase were all up-regulated. However, the proteins SAL1 phosphatase-like, phosphatidylinositol-bisphosphatase, phosphoserine phosphatase, phosphoglycerate mutase, 2-isopropylmalate synthase, and fructose-bisphosphate aldolase were down-regulated by infection stress. Fourteen identified DEPs were functionally categorized as being involved in the defense response, including molecular chaperone, redox homeostasis, and other stress related proteins. Heat shock proteins (HSP20) and FK506-binding protein 1 (protein folding chaperons) were found to be significantly up regulated between control and infected *P. yezoensis* samples. Antioxidant enzymes such as catalase, quinone oxidoreductase, aldehyde dehydrogenase, and polyadenylate-binding protein were also found to be similarly up-regulated (Fig. 4b).

Numerous proteins related to signal transduction were up-regulated such as calcium-binding protein, MAPK, and endoplasmin-like proteins (Additional file 1: Figure S3). In addition, 19 DEPs related to energy metabolism and photosynthesis were identified. Among these, the phycocyanin alpha-subunit, phycocyanobilin lyase, and the R-phycoerythrin gamma subunit were down regulated as a result of infection stress. Other down-regulated proteins included 2-isopropylmalate synthase, sulfite oxidase, the MFS transporter, ferredoxin nitrite reductase, bisphosphate nucleotidase, phosphoserine phosphatase, ribose-5-phosphate isomerase, and phosphoglycerate mutase (Additional file 1: Figure S4).

### Transcript profiling of selected genes via RT-qPCR

**Fig. 5:**
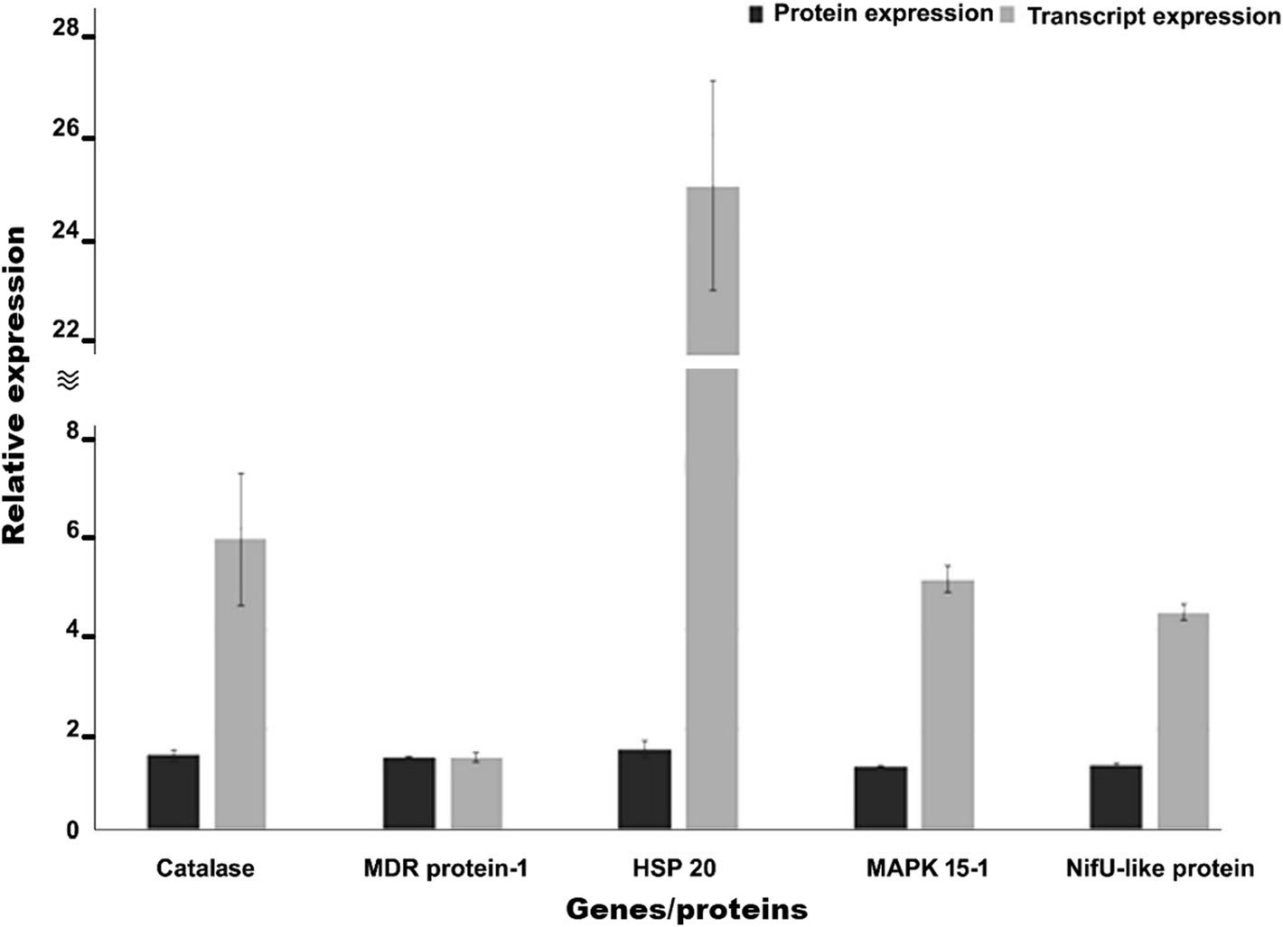
Relative expression of the differentially expressed genes selected for RT-qPCR analysis

The transcript levels of genes coding for five of the up-regulated proteins were assessed by RT-qPCR (details are provided in Table 2 and Fig. 5). These genes were chosen because they were predicted to have a role in the resistance to pathogens [50–53]. Our results showed that the PCR amplification efficiency of all the genes ranged from 91 to 100.6, and the R^2^ values ranged between 0.98 and 0.999 for all genes. The minimum value for the slopes of the standard curves was − 3.30 for the nitrogen fixation protein (Nifu) gene and the maximum recorded slope value was − 3.56 for the heat shock gene. The UBC and elf genes were used as internal controls. The 2^–ΔΔCT^ method and a paired t-test were performed on the relative expression levels of all the up-regulated genes to determine if the changes were significant. The data showed that the heat shock gene was significantly highly expressed (*P* < 0.05), with a fold change of 24.96. The other four genes were also significantly up-regulated (catalase with a fold change of 5.79, multidrug resistant with a fold change of 1.42, MAPK with a fold change of 4.99, and the NifU-like protein with a fold change of 4.32).

**Table 2:** Genes and primers used in this study for RT-qPCR expression analysis

Gene Id	Annotation	Primers, forward/reverse (5′-3′)	Product (bp)
py08174.t1	Catalase	CTTCTCCACCGTCATCCACTCC GCCGACTAGGTCCCATACACCG	118
py11399.t1	Multi-drug resistant protein	CTTCCAGCAGATGCTCACAACC TAGTAGCCAAAGCCAATCGGGA	111
py04674.t1	Heat shock protein HSP-20	GCTCGCCTACGGCTCCTTCTCT TCCACCTTGACCTTGGGCACAG	129
py09687.t1	Mitogen activated protein kinase (MAPK 15–1)	GTACGTGGCTATCAAGGGCATT TCAAGATACATCAGGTCGGGGT	120
py11267.t1	nifU-like protein	GAGGGAGTGCTCAACGAGGTGC GAGCCCTCCATCTTGAGTCGCA	116
FJ407185.1	Elongation factor-alpha	TTTCCAAGGTGCTCCTCTCCATC CGTCTCTTCATAGCGACTGCGGTT	116
Fj232910.1	Ubiquitin conjugating enzyme	GCTTTCTGTCTGGACGAGG TCTTCACAAGGATGCGGAT	181

## Discussion

This study was based on a real host–pathogen interaction namely between *P. yezoensis* (the host) and *P. porphyrae* (the pathogen). The study provided us with a composite, combined image of the response to an infection that comprised stress markers in *P. yezoensis* including oxidative stresses and metabolic processes. Using the iTRAQ technology, 762 DEPs were identified using a differential expression analysis between non-infected and infected *P. yezoensis*. The identity and function of these proteins provide a new understanding of the mechanisms of how *P. yezoensis* responds to infection stress.

### Carbohydrate metabolism

Infection stress has previously been shown to cause changes in carbohydrate metabolism and an increased cellular uptake of glucose [54, 55]. In plants, it has been shown that changes in carbohydrate metabolism are often initiated in response to stress conditions [56]. In this study, we investigated changes in the expression of proteins related to carbohydrate metabolism in the macroalgae *P. yezoensis* following infection stress. Glycolysis is a metabolic pathway that oxidizes glucose to generate ATP, and accordingly the protein levels of glycolysis-related enzymes were found to be increased, including glucose-6-phosphate isomerase, triosephosphate isomerase, and UDP-glucose 6-dehydrogenase. The enzyme fructose-bisphosphate aldolase is found in plants and plays an important role in the Calvin cycle. It has also been previously shown to be down-regulated in response to desiccation stress in *Pyropia haitanensis* [57]. Its down-regulation in this study suggests that the blades of *P. yezoensis* repress carbon fixation, since they either need more energy to fight against the disease stress, or require defense mechanisms to overcome the stress.

### Chaperone proteins and reactive oxygen species scavenging

Heat shock proteins have been shown to be highly differentially expressed in earlier proteomic research studies conducted on macroalgae, including *Ec. siliculosus* [58], and the kelp species *Laminaria digitata* [59]. They function mainly as molecular chaperones to ensure that correct protein folding occurs following exposure to a variety of metabolic stresses, including oxidative stress. Both acute and chronic oxidative stresses induce HSP responses [60–62]. The high levels of expression of heat shock proteins in infected *P. yezoensis* blades likely prevents the denaturing of other proteins affected by infection stress, and consequently is evidence of a mechanism of resistance against infection.

Infection stress can also disrupt cellular redox homeostasis and encourage the over-production of ROS. To overcome these oxidative stresses, cells have a well-developed antioxidant system [50, 63–65] that produces scavenging enzymes, such as catalase and quinone oxidoreductase. Transgenic tobacco plants with repressed catalase levels produce higher ROS levels in response to biotic and abiotic stresses [52]. The high levels of expression in infected *P. yezoensis* blades suggests that they play an important role in the defense mechanism of cells under infection stress.

Following biotic and abiotic stresses, aldehydes are known to accumulate in plants and cause damage to cell membranes through a peroxidation chain reaction. Aldehydes might also directly destroy proteins and nucleic acids, inhibiting their normal function, leading to cell death. Aldehyde dehydrogenase proteins (ALDHs) are responsible for converting aldehydes into carboxylic acids, thus reducing the peroxidation of lipid in the cell membranes. The up-regulation of both aldehyde dehydrogenase and polyadenylate-binding protein suggest their essential roles in maintaining aldehyde homeostasis and protecting cells against biotic stresses.

Derlin-1, actin, and the U6 snRNA-associated Sm-like protein were identified and shown to be down-regulated in response to infection. Actin is vital for many cellular processes since it facilitates connections with cellular membranes [66]. The U6 snRNA-associated Sm-like protein also acts as a chaperone and is involved in RNA processing, promoting the association of RNA processing factors with their substrates. The defense response of *P. yezoensis* to infection stress is complex, however, molecular chaperones, antioxidants, and stress-related proteins work collectively to protect *P. yezoensis* against infection and maintain cellular and redox homeostasis.

### Defense response and signal transduction

Plant cells sense signals upon stress and transfer these signals to the cell machinery in order to trigger an adaptive response [67]. Like the initial infection, the stress signals stimulate the transcription/translation control system and activate stress responsive mechanisms to restore damaged proteins and reinstate homeostasis. In the present study, calcium-binding protein, the WD repeat-containing protein, inositol 1,4,5-trisphosphate, endoplasmin-like protein, ubiquitin, and catalase were all found to be up regulated following infection stress. Inositol is the main constituent of the phosphoinositide pathway, and is necessary for plants in varying environments [68, 69]. The high levels of expression of this gene play a significant role in the signal transduction processes that occur in *P. yezoensis* following infection stress.

MAP kinase 15–1 was also found to be significantly and highly up regulated in our study. MAPK cascades play an important role in cellular responses to extracellular signals including stress signaling. MAPKs are central to in the protein kinase cascade, allowing these signaling pathways to spread, amplify, and integrate signals from several kinds of stimuli thereby provoking proper physiological responses, including inflammatory responses, cellular proliferation, and apoptosis [51]. Stress signaling by these genes under stress conditions confirms their role in *P. yezoensis* signal transduction and the activation of the stress responsive mechanisms to defend cells, restore damaged proteins, and reinstate homeostasis.

### Photosynthesis-related proteins and energy metabolism

Light absorption is the primary step in photosynthesis. The light harvesting system in *P. yezoensis* consists of phycobilisomes (PBSs), made up of linker polypetides and phycobiliproteins (PBPs) [70]. These PBPs are found in red algae, cyanobacteria, and cryptomonads [71] and are comprised mainly of three major proteins, phycoerythrin, phycocyanin, and allophycocyanin. Energy from sunlight is transferred initially to phycocyanin after its absorbance by phycoerythrin, before finally being transferred to chlorophyll through allophycocyanin [72]. The down-regulation of both phycocyanin alpha-subunit phycocyanobilin lyase and the R-phycoerythrin gamma subunit suggests that their ability to transfer light is inhibited. The down-regulation of most of the photosynthesis related proteins following infection stress in our study reveals the sensitivity of photosynthesis to infection stress. Fructose-1,6-bisphosphatase and fructose-bisphosphate aldolase, which play key roles in the Calvin cycle and glycolysis [57] were also down regulated in infected algal cells, which further indicates that infection stress inhibits photosynthesis in *P. yezoensis*. Therefore, based on the expression of genes related to photosynthesis, it is concluded that *P. yezoensis* decreases its photosynthetic rate upon infection stress to restrict the damage to a curable stage.

## Conclusion

This study on the *Pyropia-Pythium* host pathogen interactions gives a novel understanding into the host defense response, connecting our results with earlier studies on biotic stresses [60, 73]. This investigation of the mechanism of *P. yezoensis* resistance to *P. porphyrae* infection will shed more light on defense mechanism in macroalgae. This is the first time iTRAQ has been used to investigate proteomic expression changes in *P. yezoensis* following infection with the oomycetes pathogen *P. porphyrae*.

A number of stress-responsive proteins highlighted in this study were identified from a comparative proteomic analysis of *P. yezoensis* using the iTRAQ-based proteomic technique. A large number of the DEPs and genes detected were involved in disease stress, carbohydrate metabolism, photosynthetic activity, redox homeostasis, cell signaling, and energy metabolism as annotated by KEGG pathways and the GO database. The data showed that *P. yezoensis* resists infection by inhibiting photosynthesis, and energy and carbohydrate metabolism, as supported by the change in levels of expression of proteins involved in these processes. Thus, the current study could assist in a better understanding of the mechanisms behind infection resistance in *P. yezoensis* and improve the breeding of *P. porphyrae* -infection tolerant macroalgae.

## Material and methods

### Cultivation of *P. yezoensis*, *P. porphyrae,* and the production of *Pythium* spores

*P. yezoensis* was cultured at 10 °C using a 12 L:12D photocycle under florescent light with an intensity of 80 μmol_·_s^− 1^_·_m^− 2^. *P. porphyrae* (NBRC23353) was obtained from the Biological Resource Center of Japan and maintained on cornmeal seawater agar (CMSA) [74]. Agar discs were transferred to the liquid culture medium under axenic conditions for 7 days at 24 °C to expand the mycelia; 10 mM CaCl_2_ was added to the seawater to release the zoospores, as described previously [8].

### Infection of healthy *P. yezoensis* blades with oomycete zoospores

*P. yezoensis* blades were infected with zoospores by mixing the spore solution and healthy *Pyropia* blades; culture bottles were kept in the shaking incubator at 15 °C with a 12 L:12D photocycle under florescent light with an intensity of 80 μmol_·_s^− 1^_·_m^− 2^. Samples were monitored under the microscope every hour for the appearance of an infection, which could be readily identified on the third day of infection.

### Collection of samples

Samples were collected after the degree of infection was maximal at day eight, as assessed under the microscope, where approximately 80% of the algal cells were infected (Additional file 1: Figure S1). Following this, 0.5 g of each sample was dried on filter paper, weighed and kept at − 80 °C before further analysis. Both infected and uninfected *P. yezoensis* samples were collected in triplicate.

### Protein extraction

Protein samples were prepared as previously described [75]. Briefly, the blade samples were frozen using liquid nitrogen and then ground with a precooled pestle and mortar. A mixture of TCA/acetone (1:9) was then added five times to the powder and blended well by vortexing. The blended mix was then kept at − 20 °C for 4 h and centrifuged at 4 °C for 40 min at 6000×*g*. The supernatant was then removed and the pellet was rinsed three times using the addition of pre-cooled acetone. The precipitate was then air-dried; SDT buffer (approximately 30 (*v*/v)) was then mixed with 20–30 mg of powder, blended, and the mixture boiled for 5 min. To ensure efficient extraction, following sonication, the lysate was boiled again for 15 min, and then centrifuged at 14000×*g* for 40 min at 4 °C. The supernatant was collected, and filtered with a 0.22 μm size filter and the protein content quantified with the BCA Protein Assay Kit (Bio-Rad, USA). Finally, the sample was stored at − 80 °C before further analysis.

### SDS-PAGE separation

Proteins were isolated and visualized following electrophoresis on SDS-PAGE gels. Prior to electrophoresis, samples (20 μg of protein) were mixed with 5 × loading buffer solution, mixed well, and boiled for 5 min. Electrophoresis was carried out using a 12.5% SDS-PAGE gel. Protein bands were visualized using Coomassie Blue R-250 staining (Additional file 1: Figure S2).

### Filter-aided sample preparation (FASP digestion) and iTRAQ labeling

Samples were prepared for digestion, as previously described [76]. Protein (200 μg) present in each sample was added to 30 μL of SDT buffer (4% SDS, 100 mM DTT, 150 mM Tris-HCl pH 8.0). Low-molecular-weight components such as detergents, DTT, as well as others, were removed using UA buffer (8 M Urea, 150 mM Tris-HCl, pH 8.0) using the repeated ultrafiltration method (Microcon units, 10 kD). Following this 100 μL of iodoacetamide (100 mM IAA in UA buffer) was added to the samples and they were incubated for 30 min in the dark to avoid inhibition by the ultraviolet rays present in light. The filters were then washed three times with 100 μL of UA buffer, followed by two times with 100 μL of dissolution buffer (DS buffer). Finally, 4 μg of trypsin in 40 μL of DS buffer was used to digest the protein suspension overnight at 37 °C, and the resulting peptides were obtained as a filtrate. After trypsin digestion, the filtrate samples were analyzed using MALDI-TOF/TOF to confirm complete digestion.

Using the iTRAQ reagent 8plex Multiplex Kit, 100 μg of the peptide mixture from each sample was iTRAQ-labeled according to the manufacturer’s instructions (Applied Biosystems). The AKTA Purifier system (GE Healthcare) was used to fractionate the labeled peptides by SCX chromatography. Acidification of the dried peptide mixture was performed using buffer A (10 mM KH_2_PO_4_ in 25% of ACN, pH 3.0) and loaded onto a Poly Sulfoethyl 4.6 × 100 mm column (5 μm, 200 Å, PolyLC Inc., Maryland, USA). The peptides were eluted at a flow rate of 1 mL/min with buffer B (500 mM KCl, 10 mM KH_2_PO_4_ in 25% of ACN, pH 3.0) for 22 min, 8–52% buffer B from 22 to 47 min, 52–100% buffer B from 47 to 50 min, 100% buffer B from 50 to 58 min, after which buffer B was returned to 0% after 58 min. The elution absorbance was observed at 214 nm, and fractions were collected every 1 min. Salt was removed from the peptides fractions using C18 Cartridges (Empore™ SPE Cartridges C18 (standard density)) and concentrated by vacuum centrifugation. Protein digestion and iTRAQ labeling was carried using three replicates.

### LC-MS/MS analysis

Each fraction (10 μL) was analyzed using nano LC-MS/MS. The peptides were loaded onto a reverse phase trap column connected to a C18-reverse phase analytical column (Thermo Scientific Easy Column, 10 cm long, 75 μm inner diameter, 3 μm resin) in buffer A (0.1% formic acid), and separated using a linear gradient of buffer B (84% acetonitrile, 0.1% formic acid) at a flow rate of 300 nL/min, monitored using IntelliFlow technology. The fractions were then analyzed using a Q-Exactive mass spectrometer (Thermo Scientific) attached to an Easy nLC (Proxeon Biosystems, now Thermo Fisher Scientific) for 60/120/240 min. MS data was obtained using a data-dependent top10 method dynamically choosing the top abundant precursor ions with a m/z (300–1800 m/z) for HCD fragmentation. Resolution of the HCD spectra was set to 17,500 at m/z 200, with an isolation width of 2 m/z. The peptide recognition mode was enabled when the instrument was run.

### Data analysis

For iTRAQ protein identification, the MS/MS spectra were assessed using the MASCOT engine (Matrix Science, London, UK; version 2.2) implanted in Proteome Discoverer 1.4 querying the *Pyropia_*UniGene database [47]. The following parameters were used in the study: digestion enzyme trypsin; carbamidomethyl (C), iTRAQ4/8plex (N-term), iTRAQ 4/8plex (K) fixed modifications; oxidation (M), and iTRAQ 4/8plex (Y), variable modifications; the peptide mass tolerance was ±20 ppm; the fragment mass tolerance level was 0.1 Da; maximum missed cleavages was 2 and the peptide FDR was ≤0.01.

### Bioinformatics analysis

#### Gene ontology and KEGG **a**nnotation

Functional annotation and enrichment analyses of the DEPs in the GO database was performed by Blast2GO [77] (Version 3.3.5). Annotation of metabolic pathways was performed through blasting of the protein sequences against the online KEGG (Kyoto Encyclopedia of Genes and Genomes) database (http://geneontology.org/). The KO IDs that were retrieved were subsequently mapped to pathways in KEGG [78]. An enrichment analysis defined the protein’s role in three domains: biological process, cellular component, and molecular function. If the *P* value was under 0.05, the GO term or pathway was considered to have a significant enrichment of the different proteins.

### Hierarchical clustering

The relative protein expression data was used to perform a hierarchical clustering analysis. The omicshare online software tool (http://www.omi cshare.com/tools/home/soft/index?l=en-us), was used for this purpose. A heat map is often presented as a visual aid in addition to the dendrogram. The data are available via the ProteomeXchange, the PRIDE [79] with the identifier PXD009363.

### Quantification of transcript levels using reverse transcriptase quantitative (RT-qPCR)

The five differentially expressed genes selected for RT-qPCR analysis were first amplified, cloned, sequenced, and the plasmids purified to generate a standard curve. The reaction mixture (20 μL) included SYBR Premix (10 μL), forward and reverse primers (0.4 μL each), purified H_2_O (7.2 μL), and 2 μL of cDNA. Ubiquitin conjugating enzyme (UBC) and elongation factor-alpha (elf) genes were used as internal controls. The comparative threshold (2–ΔΔCt) method was used to calculate the fold change [80], and standard error (se) and mean Ct values were calculated. A paired t- test was performed to analyze the differential expression of the tested genes.

## Additional file

Additional file 1: **Figure S1.** *P. yezoensis* healthy cells (A) and infected (B) with *P. porphyrae* image under a light microscope using 100X lens; the red arrow represents the pathogenic oomyceteous hyphae that elongates from one cell to another. **Figure S2.** Whole cell proteins electrophoresis of Pyropia yezoensis samples. The SDS-PAGE indicating the different samples with different band size, i.e. C1-C3 representing the control samples while T1-T3 showing the treated samples with oomycetes spores. **Figure S3.** Hierarchical clustering of differentially expressed proteins under infection stress; proteins related to defense response and signal transduction. I-1 to I-3 and H-1 to H-3 the three biological replicates for infected and healthy samples, respectively. **Figure S4.** Hierarchical clustering of differentially expressed proteins under infection stress; proteins related to energy metabolism and photosynthesis. I-1 to I-3 and H-1 to H-3 the three biological replicates for infected and healthy samples, respectively. **Table S1.** Total identified 762 differentially expressed proteins along with their relative intensities in infected samples, ratios and *p*-values. (DOCX 1421 kb)

## Abbreviations

DEP: Differentially expressed proteins
GO: Gene ontology
iTRAQ: Isobaric tags for relative and absolute quantitation
KEGG: Kyoto Encyclopedia of Genes and Genomes
ROS: Reactive oxygen species
RT-qPCR: Quantitative reverse transcription PCR
