# Supplementary material for: Identification of proteins responding to pathogen-infection in the red alga Pyropia yezoensis using iTRAQ quantitative proteomics
Source: BMC Genomics. 2018 Nov 27;19:842. doi: 10.1186/s12864-018-5229-1 (PMC6260746; doi:10.1186/s12864-018-5229-1)
Supplement: Supplementary file 1 — Figure S1. P. yezoensis healthy cells (A) and infected (B) with P. porphyrae image under a light microscope using 100X lens; the red arrow represents the pathogenic oomyceteous hyphae that elongates from one cell to another. Figure S2. Whole cell proteins electrophoresis of Pyropia yezoensis samples. The SDS-PAGE indicating the different samples with different band size, i.e. C1-C3 representing the control samples while T1-T3 showing the treated samples with oomycetes spores. Figure S3. Hierarchical clustering of differentially expressed proteins under infection stress; proteins related to defense response and signal transduction. I-1 to I-3 and H-1 to H-3 the three biological replicates for infected and healthy samples, respectively. Figure S4. Hierarchical clustering of differentially expressed proteins under infection stress; proteins related to energy metabolism and photosynthesis. I-1 to I-3 and H-1 to H-3 the three biological replicates for infected and healthy samples, respectively. Table S1. Total identified 762 differentially expressed proteins along with their relative intensities in infected samples, ratios and p-values. (DOCX 1421 kb) [file 12864_2018_5229_MOESM1_ESM.docx]

**Additional file 1**

**Identification of proteins responding to pathogen-infection in the red alga *Pyropia yezoensis* using iTRAQ quantitative proteomics**

Sohrab Khan^1,2^, Yunxiang Mao^1,2,3^, Dong Gao^1,2^, Sadaf Riaz^1,2^, Zeeshan Niaz^1,2^, Lei Tang^1,2^_,_ Sohaib Khan ^1,2^, Dongmei Wang^1,2,*^

^1^Key Laboratory of Marine Genetics and Breeding (Ocean University of China), Ministry of Education, Qingdao 266003, China;

^2^Laboratory for Marine Biology and Biotechnology, Qingdao National Laboratory for Marine Science and Technology, Qingdao 266237, China;

^3^College of Marine Life Sciences, Ocean University of China, Qingdao 266003, China

^*^Email: [wangdm HYPERLINK "mailto:wangdm@ouc.edu.cn"@ HYPERLINK "mailto:wangdm@ouc.edu.cn"ouc.edu.cn](mailto:wangdm@ouc.edu.cn)

Phone number: +86 15864291929

Fax number: +86 532-82031809

**
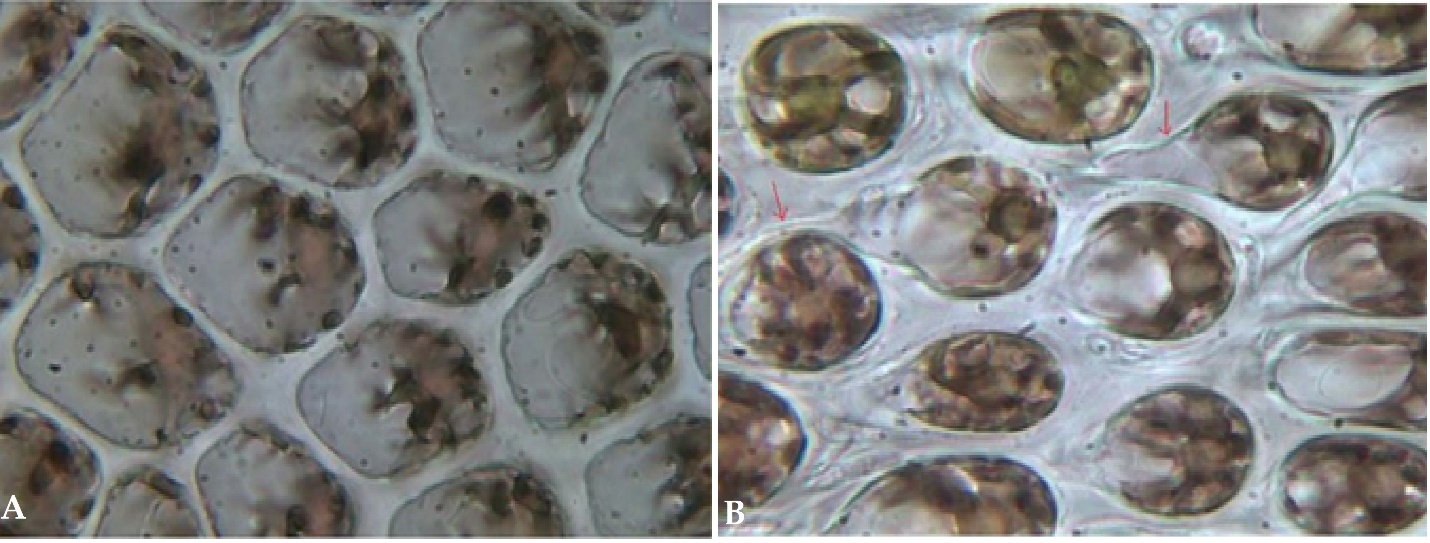
**

**Figure S1.** P. yezoensis healthy cells (A) and infected (B) with P. porphyrae image under a light microscope using 100X lens; the red arrow represents the pathogenic oomyceteous hyphae that elongates from one cell to another.

**
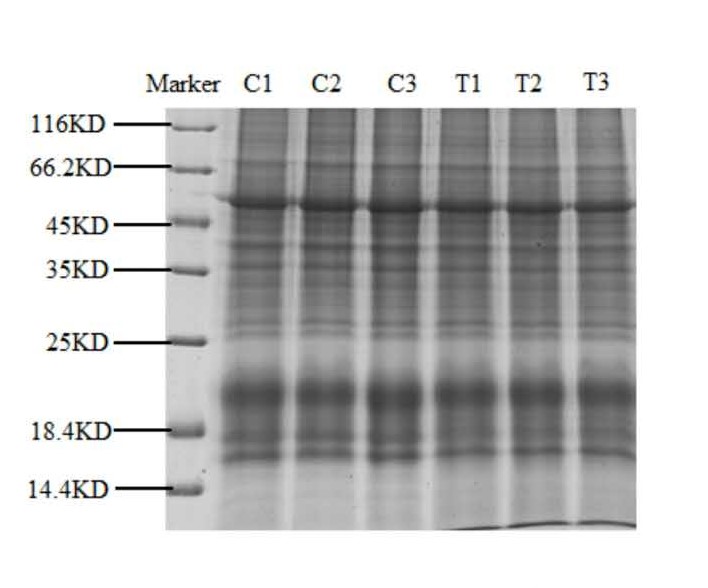
**

**Figure S2.** Whole cell proteins electrophoresis of Pyropia yezoensis samples. The SDS-PAGE indicating the different samples with different band size, i.e. C1-C3 representing the control samples while T1-T3 showing the treated samples with oomycetes spores.


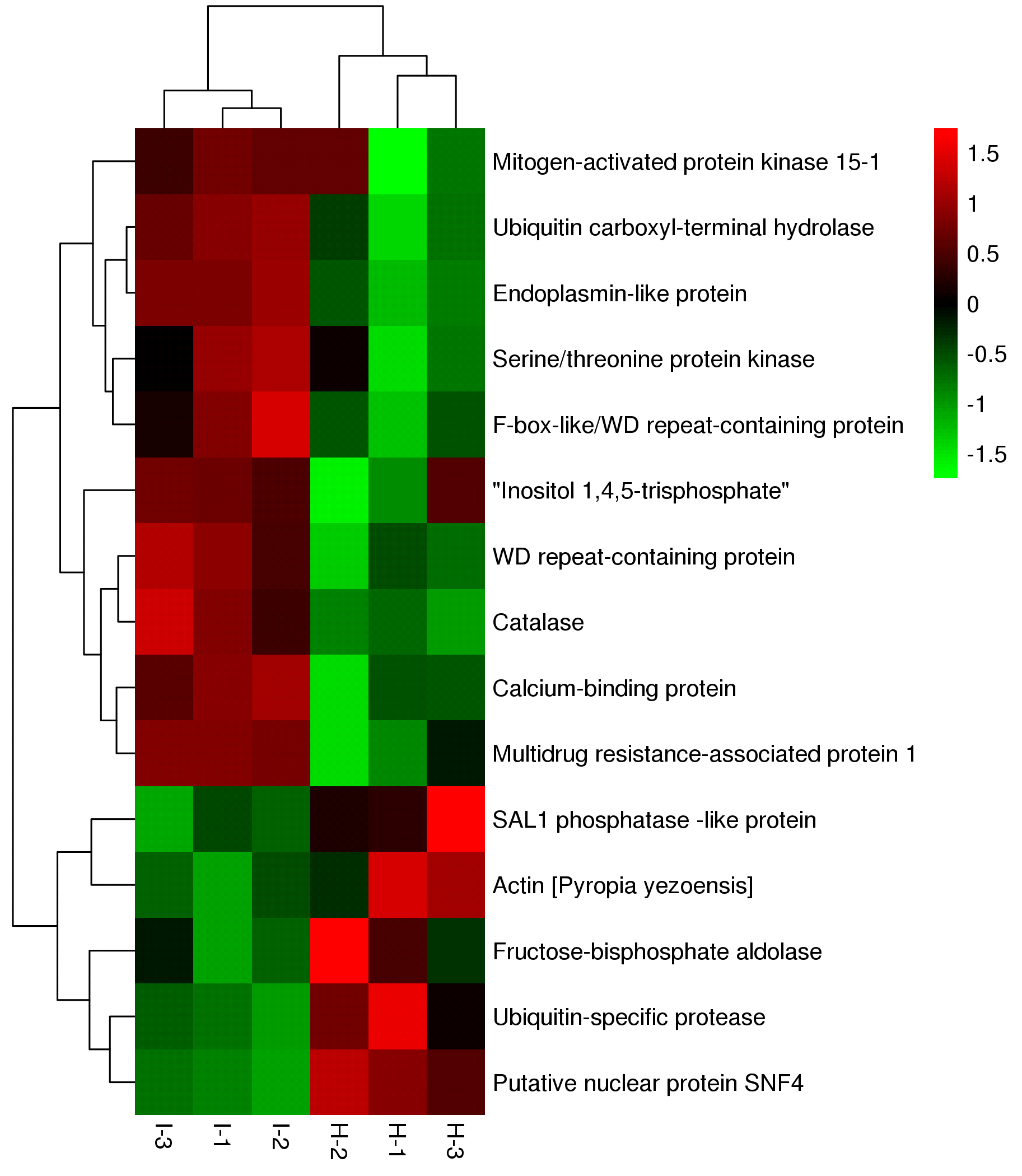


**Figure S3.** Hierarchical clustering of differentially expressed proteins under infection stress; proteins related to defense response and signal transduction. I-1 to I-3 and H-1 to H-3 the three biological replicates for infected and healthy samples, respectively.


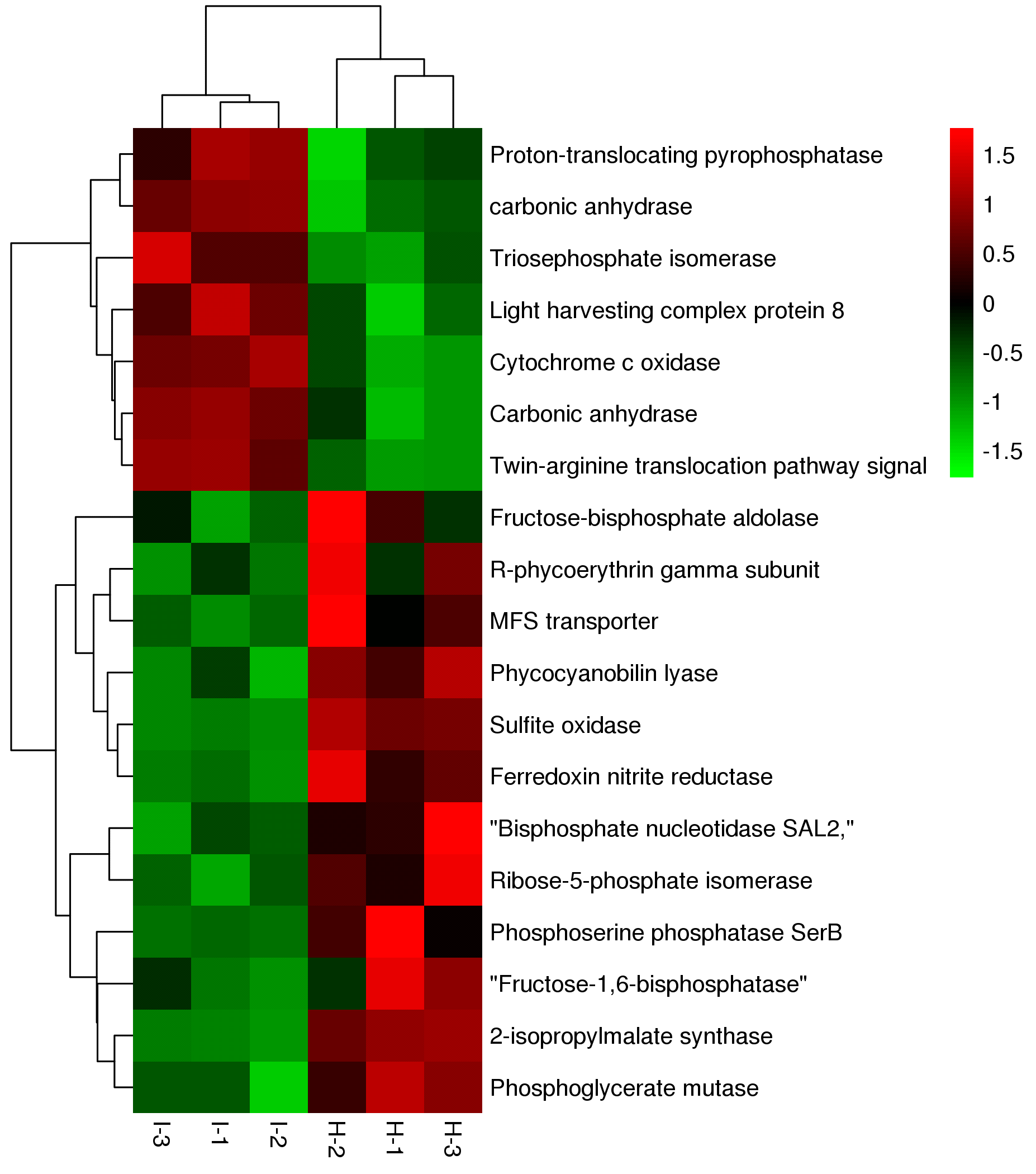


**Figure S4.** Hierarchical clustering of differentially expressed proteins under infection stress; proteins related to energy metabolism and photosynthesis. I-1 to I-3 and H-1 to H-3 the three biological replicates for infected and healthy samples, respectively.

**Table S1**

Total identified 762 differentially expressed proteins along with their relative intensities in infected samples, ratios and p-values.

| Accession | Description | I-1/H | I-2/H | I-3/H | FC(average I/H) | t test p-value |
| --- | --- | --- | --- | --- | --- | --- |
| py08881.t1 | py08881.t1 | 1.21112 | 1.210883 | 1.208886 | 1.210296574 | 1.14E-05 |
| py01087.t1 | py01087.t1 | 1.263174 | 1.264564 | 1.2583 | 1.262012833 | 5.25E-05 |
| py09529.t1 | py09529.t1 | 1.506114 | 1.496114 | 1.493731 | 1.49865286 | 5.79E-05 |
| py05119.t1 | py05119.t1 | 1.207816 | 1.208669 | 1.213292 | 1.209925608 | 6.57E-05 |
| py08292.t1 | py08292.t1 | 1.252876 | 1.260708 | 1.255722 | 1.256435399 | 7.97E-05 |
| py10791.t1 | py10791.t1 | 1.407555 | 1.42501 | 1.410291 | 1.414285182 | 0.000171 |
| py06773.t1 | py06773.t1 | 1.866181 | 1.871256 | 1.828572 | 1.855336488 | 0.000248 |
| py06895.t1 | py06895.t1 | 1.239209 | 1.234815 | 1.226448 | 1.233490708 | 0.000257 |
| py04058.t1 | py04058.t1 | 1.403227 | 1.390933 | 1.377964 | 1.390708197 | 0.000348 |
| py04988.t1 | py04988.t1 | 1.437503 | 1.460296 | 1.433547 | 1.443781932 | 0.000353 |
| py09597.t1 | py09597.t1 | 1.197148 | 1.195799 | 1.208041 | 1.200329388 | 0.000374 |
| py11399.t1 | py11399.t1 | 1.450627 | 1.421077 | 1.440406 | 1.437369773 | 0.000392 |
| py04732.t1 | py04732.t1 | 1.315066 | 1.319973 | 1.337899 | 1.324312573 | 0.000457 |
| py10706.t1 | py10706.t1 | 1.351219 | 1.326488 | 1.329475 | 1.335727274 | 0.000538 |
| py03299.t1 | py03299.t1 | 1.462092 | 1.502328 | 1.474679 | 1.479699743 | 0.000613 |
| py09822.t1 | py09822.t1 | 1.302506 | 1.328627 | 1.305956 | 1.312362974 | 0.000687 |
| py07147.t1 | py07147.t1 | 1.402288 | 1.366179 | 1.383562 | 1.38400992 | 0.000736 |
| py05354.t1 | py05354.t1 | 1.343142 | 1.312261 | 1.330736 | 1.328712787 | 0.000744 |
| py10866.t1 | py10866.t1 | 1.385645 | 1.354749 | 1.388326 | 1.376239994 | 0.000819 |
| py08884.t1 | py08884.t1 | 1.215048 | 1.222097 | 1.238795 | 1.225313251 | 0.000975 |
| py02967.t1 | py02967.t1 | 1.221743 | 1.224452 | 1.202911 | 1.216368782 | 0.000979 |
| py02067.t1 | py02067.t1 | 1.33028 | 1.3127 | 1.294835 | 1.312605082 | 0.00107 |
| py02644.t1 | py02644.t1 | 1.675432 | 1.603549 | 1.657017 | 1.645332598 | 0.001114 |
| py11465.t1 | py11465.t1 | 2.183049 | 2.303451 | 2.178095 | 2.22153195 | 0.001124 |
| py00102.t1 | py00102.t1 | 1.313282 | 1.282396 | 1.283406 | 1.293027713 | 0.001193 |
| py02623.t1 | py02623.t1 | 1.56179 | 1.548355 | 1.613872 | 1.574672386 | 0.001207 |
| py00466.t1 | py00466.t1 | 1.265483 | 1.299858 | 1.27649 | 1.280610292 | 0.001302 |
| py09687.t1 | py09687.t1 | 1.234452 | 1.253494 | 1.265638 | 1.251194619 | 0.001303 |
| py03897.t1 | py03897.t1 | 1.217726 | 1.237198 | 1.209554 | 1.221492516 | 0.001368 |
| py08376.t1 | py08376.t1 | 1.298363 | 1.303567 | 1.268851 | 1.290260393 | 0.001384 |
| py09684.t1 | py09684.t1 | 1.268608 | 1.255887 | 1.236045 | 1.253513326 | 0.001394 |
| py09479.t1 | py09479.t1 | 1.352499 | 1.336672 | 1.308226 | 1.332465513 | 0.001514 |
| py00576.t1 | py00576.t1 | 1.282689 | 1.249652 | 1.25466 | 1.262333571 | 0.001532 |
| py10700.t1 | py10700.t1 | 1.232661 | 1.253785 | 1.267035 | 1.25116014 | 0.001584 |
| py06791.t1 | py06791.t1 | 1.197635 | 1.21675 | 1.226674 | 1.213686167 | 0.001587 |
| py06185.t1 | py06185.t1 | 1.380991 | 1.361945 | 1.418865 | 1.38726687 | 0.001861 |
| py06478.t1 | py06478.t1 | 1.340063 | 1.373991 | 1.322976 | 1.34567684 | 0.001876 |
| py09535.t1 | py09535.t1 | 1.326118 | 1.348103 | 1.378705 | 1.350975427 | 0.001882 |
| py03343.t1 | py03343.t1 | 1.479298 | 1.559823 | 1.521206 | 1.520108899 | 0.001993 |
| py07062.t1 | py07062.t1 | 1.202834 | 1.236126 | 1.2278 | 1.222253239 | 0.002019 |
| py10487.t1 | py10487.t1 | 1.487859 | 1.5704 | 1.542398 | 1.533552189 | 0.002057 |
| py07959.t1 | py07959.t1 | 1.409158 | 1.479723 | 1.46512 | 1.451333511 | 0.002263 |
| py10843.t1 | py10843.t1 | 1.286464 | 1.315173 | 1.267232 | 1.289622916 | 0.002305 |
| py10410.t1 | py10410.t1 | 1.26159 | 1.244429 | 1.221254 | 1.242424614 | 0.002316 |
| py06664.t1 | py06664.t1 | 1.29876 | 1.269078 | 1.253709 | 1.273849102 | 0.002323 |
| py01241.t1 | py01241.t1 | 1.358696 | 1.423252 | 1.406346 | 1.396098057 | 0.002372 |
| py10037.t1 | py10037.t1 | 1.202544 | 1.235748 | 1.235805 | 1.22469873 | 0.002422 |
| py03249.t1 | py03249.t1 | 1.210596 | 1.210101 | 1.243587 | 1.221428026 | 0.002495 |
| py03061.t1 | py03061.t1 | 1.296939 | 1.349336 | 1.3071 | 1.317791684 | 0.002539 |
| py03720.t1 | py03720.t1 | 2.527805 | 2.379134 | 2.646342 | 2.517760073 | 0.002584 |
| py05209.t1 | py05209.t1 | 1.204549 | 1.183681 | 1.220327 | 1.202852205 | 0.002726 |
| py10600.t1 | py10600.t1 | 1.191446 | 1.192792 | 1.224352 | 1.20286358 | 0.002797 |
| py01048.t1 | py01048.t1 | 1.581927 | 1.587532 | 1.496009 | 1.555156048 | 0.002834 |
| py01693.t1 | py01693.t1 | 1.337133 | 1.404167 | 1.360918 | 1.367405811 | 0.00284 |
| py08868.t1 | py08868.t1 | 1.462271 | 1.554019 | 1.493333 | 1.503207583 | 0.002854 |
| py09954.t1 | py09954.t1 | 1.251263 | 1.290869 | 1.246314 | 1.262815255 | 0.002866 |
| py03218.t1 | py03218.t1 | 1.377541 | 1.31893 | 1.375545 | 1.357338457 | 0.002878 |
| py05949.t1 | py05949.t1 | 1.559182 | 1.658306 | 1.562129 | 1.593205782 | 0.002999 |
| py04049.t1 | py04049.t1 | 1.256824 | 1.216236 | 1.220296 | 1.231118859 | 0.003104 |
| py09453.t1 | py09453.t1 | 1.561395 | 1.53443 | 1.463184 | 1.51966968 | 0.003163 |
| py07224.t1 | py07224.t1 | 1.296032 | 1.26697 | 1.243162 | 1.268721585 | 0.003221 |
| py08927.t1 | py08927.t1 | 1.529154 | 1.499721 | 1.603979 | 1.544284756 | 0.003235 |
| py07138.t1 | py07138.t1 | 1.339874 | 1.279174 | 1.321204 | 1.313417219 | 0.003264 |
| py10107.t1 | py10107.t1 | 1.181026 | 1.205168 | 1.220991 | 1.202395224 | 0.00328 |
| py06425.t1 | py06425.t1 | 1.241635 | 1.239424 | 1.284534 | 1.255197813 | 0.003294 |
| py08816.t1 | py08816.t1 | 1.54499 | 1.45295 | 1.538114 | 1.512018136 | 0.003326 |
| py01055.t1 | py01055.t1 | 1.274602 | 1.288927 | 1.237079 | 1.266869561 | 0.003339 |
| py00803.t1 | py00803.t1 | 1.610455 | 1.69734 | 1.574801 | 1.627531842 | 0.003346 |
| py06341.t1 | py06341.t1 | 1.330483 | 1.357117 | 1.290514 | 1.326038025 | 0.003505 |
| py07400.t2 | py07400.t2 | 1.265045 | 1.277758 | 1.226909 | 1.256570851 | 0.003527 |
| py06538.t1 | py06538.t1 | 1.369379 | 1.321043 | 1.303243 | 1.331221541 | 0.00354 |
| py03912.t1 | py03912.t1 | 1.222057 | 1.200799 | 1.180482 | 1.201112652 | 0.003543 |
| py05413.t1 | py05413.t1 | 1.256166 | 1.27271 | 1.221293 | 1.25005639 | 0.003652 |
| py02931.t1 | py02931.t1 | 1.51859 | 1.436363 | 1.431911 | 1.462288113 | 0.003695 |
| py10113.t1 | py10113.t1 | 1.273994 | 1.22109 | 1.253373 | 1.249485689 | 0.003786 |
| py05278.t1 | py05278.t1 | 1.258794 | 1.317065 | 1.272817 | 1.282891907 | 0.003831 |
| py09483.t1 | py09483.t1 | 1.21356 | 1.26493 | 1.247871 | 1.242120339 | 0.00387 |
| py09468.t1 | py09468.t1 | 1.342634 | 1.42214 | 1.408514 | 1.391095926 | 0.003917 |
| py06130.t1 | py06130.t1 | 1.262029 | 1.280777 | 1.225611 | 1.256138838 | 0.003974 |
| py04289.t1 | py04289.t1 | 1.385831 | 1.449416 | 1.364353 | 1.399866623 | 0.004054 |
| py00044.t1 | py00044.t1 | 1.680625 | 1.573072 | 1.558174 | 1.603957292 | 0.004055 |
| py06315.t1 | py06315.t1 | 2.031217 | 2.053315 | 1.854883 | 1.97980513 | 0.004081 |
| py08137.t1 | py08137.t1 | 1.482594 | 1.524255 | 1.41871 | 1.475186439 | 0.004146 |
| py01158.t1 | py01158.t1 | 1.351939 | 1.281238 | 1.314445 | 1.315874119 | 0.004154 |
| py10389.t1 | py10389.t1 | 1.243967 | 1.214889 | 1.269772 | 1.242875879 | 0.004233 |
| py03213.t1 | py03213.t1 | 1.328919 | 1.387619 | 1.412234 | 1.376257562 | 0.004286 |
| py00610.t1 | py00610.t1 | 1.389693 | 1.333105 | 1.313137 | 1.34531147 | 0.004379 |
| py07072.t1 | py07072.t1 | 1.242094 | 1.28608 | 1.230552 | 1.252908923 | 0.004444 |
| py04210.t1 | py04210.t1 | 1.3599 | 1.425153 | 1.340892 | 1.375314874 | 0.00459 |
| py06004.t1 | py06004.t1 | 1.230172 | 1.291366 | 1.254133 | 1.258556904 | 0.004708 |
| py04439.t1 | py04439.t1 | 1.469257 | 1.549342 | 1.596394 | 1.538331192 | 0.004719 |
| py08281.t1 | py08281.t1 | 1.212788 | 1.222923 | 1.265635 | 1.233781906 | 0.004764 |
| py06003.t1 | py06003.t1 | 1.220829 | 1.207258 | 1.173925 | 1.200670294 | 0.004788 |
| py04357.t1 | py04357.t1 | 1.393166 | 1.343845 | 1.309094 | 1.348701283 | 0.004857 |
| py09434.t1 | py09434.t1 | 1.260084 | 1.282178 | 1.330296 | 1.290852761 | 0.00504 |
| py06569.t1 | py06569.t1 | 1.273249 | 1.327116 | 1.259949 | 1.286771041 | 0.005088 |
| py06607.t1 | py06607.t1 | 1.194974 | 1.237281 | 1.190531 | 1.207595347 | 0.005111 |
| py00196.t1 | py00196.t1 | 1.358374 | 1.276938 | 1.322884 | 1.319398646 | 0.005403 |
| py00010.t1 | py00010.t1 | 1.589493 | 1.511271 | 1.457395 | 1.519386138 | 0.005407 |
| py11428.t1 | py11428.t1 | 1.20148 | 1.258398 | 1.248556 | 1.236144618 | 0.005486 |
| py05937.t1 | py05937.t1 | 1.438574 | 1.351549 | 1.355286 | 1.381802953 | 0.00549 |
| py06616.t1 | py06616.t1 | 1.488974 | 1.543073 | 1.63064 | 1.554228955 | 0.0055 |
| py03517.t1 | py03517.t1 | 1.336962 | 1.40569 | 1.318497 | 1.353716612 | 0.005578 |
| py07136.t2 | py07136.t2 | 1.231108 | 1.239992 | 1.186655 | 1.21925194 | 0.005615 |
| py03770.t1 | py03770.t1 | 1.894577 | 1.882517 | 1.70176 | 1.826284537 | 0.005648 |
| py00323.t1 | py00323.t1 | 1.419908 | 1.358512 | 1.324786 | 1.3677351 | 0.005684 |
| py07140.t1 | py07140.t1 | 1.242064 | 1.303375 | 1.243632 | 1.263023798 | 0.005835 |
| py07613.t1 | py07613.t1 | 1.370716 | 1.329132 | 1.282984 | 1.327610787 | 0.005928 |
| py07638.t1 | py07638.t1 | 1.338971 | 1.359269 | 1.275781 | 1.32467335 | 0.005942 |
| py08695.t1 | py08695.t1 | 1.494992 | 1.447069 | 1.581636 | 1.507898947 | 0.005958 |
| py03317.t1 | py03317.t1 | 1.208236 | 1.257726 | 1.203195 | 1.223052498 | 0.006029 |
| py02276.t1 | py02276.t1 | 1.337167 | 1.416842 | 1.327939 | 1.360649459 | 0.006068 |
| py00961.t1 | py00961.t1 | 1.376891 | 1.290591 | 1.3647 | 1.344060908 | 0.006086 |
| py07981.t1 | py07981.t1 | 1.327803 | 1.43272 | 1.395052 | 1.385191602 | 0.006286 |
| py02012.t1 | py02012.t1 | 1.606649 | 1.805991 | 1.73361 | 1.71541673 | 0.006566 |
| py01269.t1 | py01269.t1 | 1.571555 | 1.705848 | 1.545845 | 1.607749399 | 0.006597 |
| py01967.t1 | py01967.t1 | 1.516002 | 1.388038 | 1.470379 | 1.45813946 | 0.006614 |
| py08225.t1 | py08225.t1 | 1.739178 | 1.837722 | 1.629243 | 1.735380839 | 0.006638 |
| py08163.t1 | py08163.t1 | 1.7443 | 1.591037 | 1.58109 | 1.638808835 | 0.006768 |
| py10082.t2 | py10082.t2 | 1.445408 | 1.336481 | 1.371852 | 1.384580174 | 0.006887 |
| py06686.t1 | py06686.t1 | 1.171872 | 1.22551 | 1.221292 | 1.206224683 | 0.0069 |
| py03334.t1 | py03334.t1 | 1.241471 | 1.214763 | 1.179713 | 1.211982346 | 0.007041 |
| py08636.t1 | py08636.t1 | 1.385454 | 1.353102 | 1.286806 | 1.341787434 | 0.007139 |
| py10784.t1 | py10784.t1 | 1.322571 | 1.432736 | 1.401355 | 1.385553861 | 0.007146 |
| py05788.t1 | py05788.t1 | 1.270636 | 1.292788 | 1.217566 | 1.260330174 | 0.007269 |
| py08519.t1 | py08519.t1 | 1.291566 | 1.378478 | 1.299194 | 1.323079459 | 0.007316 |
| py00251.t1 | py00251.t1 | 1.216296 | 1.177348 | 1.239676 | 1.211106489 | 0.007334 |
| py07742.t1 | py07742.t1 | 1.301977 | 1.351487 | 1.26058 | 1.3046814 | 0.007356 |
| py05285.t1 | py05285.t1 | 2.172272 | 2.361665 | 2.582153 | 2.372029762 | 0.007369 |
| py01212.t1 | py01212.t1 | 1.280861 | 1.340899 | 1.255616 | 1.29245862 | 0.007396 |
| py11267.t1 | py11267.t1 | 1.311093 | 1.285206 | 1.229253 | 1.275183907 | 0.007614 |
| py02308.t1 | py02308.t1 | 1.355796 | 1.382684 | 1.282389 | 1.340289621 | 0.007669 |
| py11032.t1 | py11032.t1 | 2.319485 | 2.213234 | 2.62812 | 2.386946445 | 0.007952 |
| py01788.t1 | py01788.t1 | 1.276346 | 1.370926 | 1.299852 | 1.315708076 | 0.008012 |
| py03437.t1 | py03437.t1 | 2.291551 | 2.507545 | 2.100132 | 2.299742475 | 0.008098 |
| py07294.t1 | py07294.t1 | 1.202249 | 1.228915 | 1.275665 | 1.235609765 | 0.008191 |
| py10617.t1 | py10617.t1 | 1.255975 | 1.186018 | 1.223408 | 1.221800102 | 0.008201 |
| py07482.t1 | py07482.t1 | 1.519416 | 1.685051 | 1.704478 | 1.636314696 | 0.008408 |
| py05697.t1 | py05697.t1 | 1.250014 | 1.256195 | 1.188447 | 1.231552108 | 0.008611 |
| py11074.t1 | py11074.t1 | 1.461465 | 1.508493 | 1.629011 | 1.532989773 | 0.008649 |
| py10645.t1 | py10645.t1 | 1.292234 | 1.210357 | 1.25711 | 1.253233802 | 0.008657 |
| py07282.t1 | py07282.t1 | 1.502575 | 1.586835 | 1.423265 | 1.504224974 | 0.008658 |
| py08024.t1 | py08024.t1 | 1.228933 | 1.250573 | 1.311589 | 1.263698467 | 0.008692 |
| py06123.t1 | py06123.t1 | 1.209854 | 1.231283 | 1.166429 | 1.202522145 | 0.008757 |
| py01532.t1 | py01532.t1 | 1.27555 | 1.361112 | 1.378925 | 1.338528923 | 0.008766 |
| py05370.t1 | py05370.t1 | 1.422201 | 1.49858 | 1.359408 | 1.426729413 | 0.008775 |
| py02872.t1 | py02872.t1 | 1.238317 | 1.170799 | 1.209765 | 1.206293649 | 0.008878 |
| py01388.t1 | py01388.t1 | 1.311118 | 1.23522 | 1.323303 | 1.289880315 | 0.008915 |
| py00242.t1 | py00242.t1 | 1.368996 | 1.320245 | 1.264035 | 1.317758411 | 0.008985 |
| py02190.t1 | py02190.t1 | 1.304813 | 1.396102 | 1.424616 | 1.375177215 | 0.009148 |
| py09098.t1 | py09098.t1 | 3.60662 | 3.753387 | 2.979055 | 3.446354201 | 0.009291 |
| py01288.t1 | py01288.t1 | 1.291621 | 1.379687 | 1.407442 | 1.359583105 | 0.009296 |
| pyi00273.t1 | pyi00273.t1 | 2.283585 | 2.528563 | 2.091587 | 2.301244553 | 0.009312 |
| py10541.t1 | py10541.t1 | 1.428096 | 1.597326 | 1.485977 | 1.503799735 | 0.009576 |
| py11251.t1 | py11251.t1 | 1.280224 | 1.197792 | 1.250619 | 1.2428784 | 0.00971 |
| py11194.t1 | py11194.t1 | 1.24724 | 1.221617 | 1.308599 | 1.259152332 | 0.009771 |
| py06907.t1 | py06907.t1 | 1.499016 | 1.478154 | 1.649631 | 1.542266712 | 0.009778 |
| py08756.t1 | py08756.t1 | 1.735332 | 1.535355 | 1.73557 | 1.668752228 | 0.009801 |
| pyi00272.t1 | pyi00272.t1 | 1.25834 | 1.242016 | 1.183319 | 1.22789189 | 0.009844 |
| py06046.t1 | py06046.t1 | 1.466729 | 1.325717 | 1.406197 | 1.399547797 | 0.010288 |
| py08593.t1 | py08593.t1 | 1.326222 | 1.233712 | 1.256104 | 1.272012831 | 0.010331 |
| py06688.t1 | py06688.t1 | 1.620079 | 1.751489 | 1.525747 | 1.632438383 | 0.010544 |
| py05353.t1 | py05353.t1 | 1.476365 | 1.370268 | 1.33982 | 1.395484373 | 0.010773 |
| py03116.t1 | py03116.t1 | 1.227325 | 1.194084 | 1.278182 | 1.233196958 | 0.010818 |
| py06712.t1 | py06712.t1 | 1.245928 | 1.238475 | 1.173332 | 1.219245005 | 0.01088 |
| py01631.t1 | py01631.t1 | 1.284351 | 1.196434 | 1.255237 | 1.245340732 | 0.010926 |
| py02495.t1 | py02495.t1 | 1.201176 | 1.274354 | 1.287277 | 1.254269221 | 0.010933 |
| py06037.t1 | py06037.t1 | 1.428174 | 1.613553 | 1.585535 | 1.542420635 | 0.011125 |
| py00603.t1 | py00603.t1 | 1.416844 | 1.48419 | 1.3323 | 1.411111204 | 0.011232 |
| py07715.t1 | py07715.t1 | 1.2354 | 1.297984 | 1.20862 | 1.247334442 | 0.011267 |
| py06292.t1 | py06292.t1 | 1.602256 | 1.507722 | 1.735145 | 1.615041418 | 0.011307 |
| py01088.t1 | py01088.t1 | 1.29655 | 1.260242 | 1.203135 | 1.253308928 | 0.011325 |
| py08622.t1 | py08622.t1 | 1.281533 | 1.278102 | 1.198493 | 1.252709111 | 0.011327 |
| py10524.t1 | py10524.t1 | 1.431382 | 1.543485 | 1.37923 | 1.451365795 | 0.01133 |
| py00401.t1 | py00401.t1 | 1.598932 | 1.442466 | 1.640742 | 1.560713114 | 0.011384 |
| py04481.t1 | py04481.t1 | 1.2648 | 1.294663 | 1.201899 | 1.253787187 | 0.011406 |
| py09005.t1 | py09005.t1 | 1.254661 | 1.222454 | 1.174168 | 1.217094376 | 0.01141 |
| py08038.t1 | py08038.t1 | 1.916842 | 1.772946 | 1.627727 | 1.772504864 | 0.011472 |
| py06990.t1 | py06990.t1 | 1.333581 | 1.279369 | 1.228255 | 1.280401842 | 0.011558 |
| py06756.t1 | py06756.t1 | 1.503426 | 1.426711 | 1.343767 | 1.424634722 | 0.011582 |
| py09293.t1 | py09293.t1 | 1.281022 | 1.19267 | 1.231503 | 1.235064968 | 0.011624 |
| py08699.t1 | py08699.t1 | 1.325084 | 1.36984 | 1.251443 | 1.315455465 | 0.011761 |
| py08925.t1 | py08925.t1 | 1.209506 | 1.303132 | 1.290576 | 1.267738134 | 0.011797 |
| py04904.t1 | py04904.t1 | 1.335123 | 1.33223 | 1.457006 | 1.374786204 | 0.011824 |
| py09691.t1 | py09691.t1 | 1.560018 | 1.643829 | 1.437006 | 1.546951057 | 0.011845 |
| py03553.t1 | py03553.t1 | 1.332879 | 1.370925 | 1.252171 | 1.318658572 | 0.011857 |
| py06467.t1 | py06467.t1 | 1.211351 | 1.20919 | 1.288477 | 1.236339323 | 0.011956 |
| py07524.t1 | py07524.t1 | 1.312018 | 1.213269 | 1.291559 | 1.272281944 | 0.011995 |
| py08012.t1 | py08012.t1 | 1.346697 | 1.264094 | 1.243515 | 1.284768562 | 0.012038 |
| py00364.t1 | py00364.t1 | 1.537714 | 1.648688 | 1.440385 | 1.542262386 | 0.012092 |
| py03303.t1 | py03303.t1 | 1.480147 | 1.405148 | 1.593637 | 1.49297742 | 0.012128 |
| py01441.t1 | py01441.t1 | 1.314227 | 1.3113 | 1.430359 | 1.351962094 | 0.012183 |
| py06634.t1 | py06634.t1 | 1.293072 | 1.331202 | 1.424553 | 1.349609118 | 0.012251 |
| py09210.t1 | py09210.t1 | 1.681895 | 1.761943 | 1.511699 | 1.651845617 | 0.012572 |
| pyi00397.t1 | pyi00397.t1 | 1.211842 | 1.302302 | 1.307922 | 1.274021771 | 0.012663 |
| py00696.t1 | py00696.t1 | 1.230405 | 1.319054 | 1.229271 | 1.259576826 | 0.012874 |
| py02519.t1 | py02519.t1 | 2.162232 | 1.801653 | 1.885721 | 1.949868696 | 0.012895 |
| py10091.t1 | py10091.t1 | 1.338533 | 1.412448 | 1.274532 | 1.341837655 | 0.013317 |
| py08088.t1 | py08088.t1 | 1.179004 | 1.260711 | 1.196688 | 1.212134225 | 0.013413 |
| py07469.t1 | py07469.t1 | 1.313389 | 1.22893 | 1.346051 | 1.29612348 | 0.013603 |
| py09509.t1 | py09509.t1 | 1.454387 | 1.455131 | 1.310481 | 1.406666148 | 0.013699 |
| py08620.t1 | py08620.t1 | 1.411101 | 1.466301 | 1.306181 | 1.394527696 | 0.013873 |
| py03874.t1 | py03874.t1 | 1.264751 | 1.404814 | 1.350712 | 1.340092576 | 0.014075 |
| py09164.t1 | py09164.t1 | 1.525958 | 1.607289 | 1.396011 | 1.509752934 | 0.014257 |
| py08256.t1 | py08256.t1 | 1.273104 | 1.366994 | 1.249685 | 1.296594197 | 0.014292 |
| py05680.t1 | py05680.t1 | 1.31011 | 1.416882 | 1.283181 | 1.336724366 | 0.014384 |
| py07233.t1 | py07233.t1 | 1.347064 | 1.424634 | 1.277749 | 1.349815867 | 0.014391 |
| py07589.t1 | py07589.t1 | 1.482902 | 1.454627 | 1.661477 | 1.53300212 | 0.014441 |
| py04403.t1 | py04403.t1 | 1.263445 | 1.281114 | 1.184653 | 1.243070713 | 0.014556 |
| py09626.t1 | py09626.t1 | 1.200587 | 1.163556 | 1.250006 | 1.204716321 | 0.014635 |
| py07459.t1 | py07459.t1 | 1.564448 | 1.595459 | 1.821175 | 1.660360297 | 0.01468 |
| py07448.t1 | py07448.t1 | 1.264198 | 1.171047 | 1.228435 | 1.221226766 | 0.014709 |
| py09720.t1 | py09720.t1 | 1.647501 | 1.802822 | 1.52324 | 1.657854297 | 0.014779 |
| py08174.t1 | py08174.t1 | 1.504895 | 1.372859 | 1.57706 | 1.48493807 | 0.014862 |
| py10966.t1 | py10966.t1 | 1.335297 | 1.217218 | 1.276565 | 1.276360392 | 0.014875 |
| py05715.t1 | py05715.t1 | 1.430113 | 1.548761 | 1.359994 | 1.446289434 | 0.014897 |
| py00670.t1 | py00670.t1 | 1.260922 | 1.237576 | 1.168873 | 1.222457005 | 0.015075 |
| py02384.t1 | py02384.t1 | 1.518668 | 1.540408 | 1.755981 | 1.605019149 | 0.015313 |
| py02157.t1 | py02157.t1 | 1.467882 | 1.33528 | 1.320328 | 1.374496479 | 0.015319 |
| py03491.t1 | py03491.t1 | 1.234635 | 1.267556 | 1.171663 | 1.224617929 | 0.015326 |
| py03062.t1 | py03062.t1 | 2.180556 | 2.222107 | 1.800435 | 2.067699575 | 0.015427 |
| py10347.t1 | py10347.t1 | 1.636138 | 1.827513 | 1.992488 | 1.818713356 | 0.015451 |
| py01925.t1 | py01925.t1 | 1.249027 | 1.319392 | 1.38798 | 1.318799684 | 0.015466 |
| py10403.t1 | py10403.t1 | 1.234265 | 1.277303 | 1.177491 | 1.229686472 | 0.015469 |
| py00461.t1 | py00461.t1 | 1.187203 | 1.26547 | 1.290967 | 1.247879892 | 0.015494 |
| py09261.t1 | py09261.t1 | 1.232382 | 1.175788 | 1.276317 | 1.228162332 | 0.015877 |
| py05793.t1 | py05793.t1 | 1.24354 | 1.304412 | 1.194382 | 1.247444533 | 0.01614 |
| py07124.t1 | py07124.t1 | 1.352384 | 1.319797 | 1.482578 | 1.384919424 | 0.016283 |
| py05035.t1 | py05035.t1 | 1.357033 | 1.226671 | 1.329509 | 1.304404574 | 0.016563 |
| py11270.t1 | py11270.t1 | 1.17418 | 1.276759 | 1.228404 | 1.226447289 | 0.016692 |
| py02236.t1 | py02236.t1 | 1.3435 | 1.414675 | 1.260137 | 1.339437303 | 0.016872 |
| py02635.t1 | py02635.t1 | 1.311127 | 1.266213 | 1.194175 | 1.257171569 | 0.017095 |
| py02045.t1 | py02045.t1 | 1.464917 | 1.658728 | 1.438015 | 1.520553257 | 0.017373 |
| py08575.t1 | py08575.t1 | 1.356629 | 1.223848 | 1.281822 | 1.28743298 | 0.017413 |
| py07050.t1 | py07050.t1 | 1.474748 | 1.622788 | 1.394546 | 1.497360699 | 0.017591 |
| py05980.t1 | py05980.t1 | 1.434607 | 1.324812 | 1.277157 | 1.34552539 | 0.01772 |
| py07923.t1 | py07923.t1 | 2.083347 | 2.54487 | 2.021522 | 2.216579808 | 0.017926 |
| py03549.t1 | py03549.t1 | 1.163679 | 1.261362 | 1.249739 | 1.224926635 | 0.018248 |
| py00485.t1 | py00485.t1 | 1.528287 | 1.546212 | 1.343452 | 1.472650135 | 0.018286 |
| py04008.t1 | py04008.t1 | 1.452336 | 1.28116 | 1.423874 | 1.385789829 | 0.018326 |
| py07105.t1 | py07105.t1 | 1.214443 | 1.291246 | 1.18399 | 1.229892879 | 0.018729 |
| py03268.t1 | py03268.t1 | 2.052241 | 2.469156 | 1.94284 | 2.154745558 | 0.018741 |
| py05891.t1 | py05891.t1 | 1.255095 | 1.158588 | 1.193717 | 1.20246643 | 0.018853 |
| py02364.t1 | py02364.t1 | 4.4318 | 5.478257 | 6.642135 | 5.517397178 | 0.019391 |
| py01366.t1 | py01366.t1 | 1.260419 | 1.208065 | 1.158019 | 1.208834399 | 0.019457 |
| py09560.t1 | py09560.t1 | 1.276317 | 1.244221 | 1.166737 | 1.229091699 | 0.019567 |
| py07924.t1 | py07924.t1 | 1.861845 | 2.002295 | 1.601694 | 1.821944631 | 0.019781 |
| py11502.t1 | py11502.t1 | 2.010115 | 2.106045 | 2.581932 | 2.232697131 | 0.019957 |
| py06501.t1 | py06501.t1 | 1.333411 | 1.237707 | 1.397139 | 1.32275224 | 0.019991 |
| py07453.t1 | py07453.t1 | 1.479664 | 1.491892 | 1.301782 | 1.424445901 | 0.020313 |
| py04789.t1 | py04789.t1 | 1.295801 | 1.295977 | 1.183361 | 1.258379808 | 0.020431 |
| py04674.t1 | py04674.t1 | 1.617345 | 1.75272 | 1.447134 | 1.605733231 | 0.020644 |
| py07850.t1 | py07850.t1 | 1.277478 | 1.404774 | 1.258148 | 1.313466781 | 0.020857 |
| py05328.t1 | py05328.t1 | 1.465265 | 1.589095 | 1.350605 | 1.468321642 | 0.020945 |
| py09511.t1 | py09511.t1 | 1.147558 | 1.210844 | 1.25034 | 1.202913976 | 0.021077 |
| py00317.t1 | py00317.t1 | 1.241054 | 1.367528 | 1.242717 | 1.283766082 | 0.021098 |
| py07833.t1 | py07833.t1 | 1.238118 | 1.146543 | 1.239223 | 1.207961205 | 0.02112 |
| py04201.t1 | py04201.t1 | 1.350356 | 1.519529 | 1.333547 | 1.401144057 | 0.021225 |
| py07285.t1 | py07285.t1 | 1.290767 | 1.485234 | 1.366561 | 1.380853833 | 0.021373 |
| py01514.t1 | py01514.t1 | 2.057076 | 2.547232 | 1.978539 | 2.194282267 | 0.021483 |
| py09406.t1 | py09406.t1 | 1.477862 | 1.4244 | 1.279714 | 1.393992074 | 0.021831 |
| py00535.t1 | py00535.t1 | 1.188643 | 1.178353 | 1.280894 | 1.215963529 | 0.022037 |
| py06063.t1 | py06063.t1 | 1.380767 | 1.228386 | 1.37532 | 1.328157747 | 0.02236 |
| py09638.t1 | py09638.t1 | 1.208807 | 1.360456 | 1.317345 | 1.295536008 | 0.022519 |
| py01723.t1 | py01723.t1 | 1.29173 | 1.253653 | 1.168689 | 1.238024264 | 0.022559 |
| py03291.t1 | py03291.t1 | 1.241982 | 1.247862 | 1.14756 | 1.212468079 | 0.022605 |
| py00855.t1 | py00855.t1 | 1.361062 | 1.453896 | 1.263063 | 1.359340247 | 0.022711 |
| py09068.t1 | py09068.t1 | 1.152719 | 1.264115 | 1.210435 | 1.209089994 | 0.022856 |
| py08584.t1 | py08584.t1 | 1.467669 | 1.32854 | 1.283508 | 1.359905714 | 0.022906 |
| py03216.t1 | py03216.t1 | 1.244426 | 1.303372 | 1.17397 | 1.240589143 | 0.023329 |
| py04788.t1 | py04788.t1 | 1.291391 | 1.270201 | 1.16865 | 1.243414282 | 0.023371 |
| py08222.t1 | py08222.t1 | 1.251012 | 1.273154 | 1.157664 | 1.227276788 | 0.023397 |
| py04079.t1 | py04079.t1 | 1.207469 | 1.252524 | 1.144194 | 1.201395903 | 0.023484 |
| py01107.t1 | py01107.t1 | 1.391023 | 1.273051 | 1.235734 | 1.299936098 | 0.023492 |
| py07795.t1 | py07795.t1 | 1.428418 | 1.406762 | 1.249306 | 1.361495349 | 0.023521 |
| py00241.t1 | py00241.t1 | 1.253989 | 1.331206 | 1.438344 | 1.341179959 | 0.023677 |
| py10798.t1 | py10798.t1 | 1.243129 | 1.143065 | 1.239552 | 1.208582223 | 0.023812 |
| py03381.t1 | py03381.t1 | 1.146359 | 1.239383 | 1.252851 | 1.21286439 | 0.023855 |
| py06883.t1 | py06883.t1 | 1.188588 | 1.284857 | 1.177169 | 1.216871477 | 0.023913 |
| py01774.t1 | py01774.t1 | 2.51956 | 3.252397 | 2.379518 | 2.717158215 | 0.023955 |
| py10101.t1 | py10101.t1 | 1.251669 | 1.172498 | 1.304733 | 1.242966231 | 0.024105 |
| py10466.t1 | py10466.t1 | 1.50031 | 1.470442 | 1.75782 | 1.576190352 | 0.024161 |
| py08929.t1 | py08929.t1 | 1.289607 | 1.428412 | 1.260467 | 1.32616185 | 0.024318 |
| py00793.t1 | py00793.t1 | 1.159155 | 1.280199 | 1.217649 | 1.219001138 | 0.024534 |
| py08228.t1 | py08228.t1 | 1.191816 | 1.273234 | 1.161586 | 1.208878575 | 0.024543 |
| py05745.t1 | py05745.t1 | 1.904066 | 1.561636 | 1.591629 | 1.68577706 | 0.024555 |
| py10304.t1 | py10304.t1 | 1.163188 | 1.269586 | 1.287134 | 1.239969061 | 0.025065 |
| py09487.t1 | py09487.t1 | 1.188292 | 1.171521 | 1.282587 | 1.214133238 | 0.025083 |
| py07478.t1 | py07478.t1 | 1.435635 | 1.642943 | 1.384931 | 1.487836382 | 0.025188 |
| py10015.t1 | py10015.t1 | 1.656196 | 1.542424 | 1.365995 | 1.521538105 | 0.025216 |
| py09167.t1 | py09167.t1 | 1.229227 | 1.262506 | 1.146577 | 1.212769844 | 0.025246 |
| py07043.t1 | py07043.t1 | 1.170254 | 1.205195 | 1.291846 | 1.222431591 | 0.025402 |
| py00509.t1 | py00509.t1 | 1.2481 | 1.14567 | 1.255075 | 1.216281736 | 0.025707 |
| py10715.t1 | py10715.t1 | 1.3607 | 1.241204 | 1.214405 | 1.272102998 | 0.026242 |
| py06206.t1 | py06206.t1 | 1.254291 | 1.455018 | 1.431885 | 1.380398162 | 0.026677 |
| py11378.t1 | py11378.t1 | 1.309313 | 1.205015 | 1.183595 | 1.232640838 | 0.026748 |
| py11528.t1 | py11528.t1 | 1.251292 | 1.397248 | 1.244741 | 1.297760201 | 0.02683 |
| py01946.t1 | py01946.t1 | 1.360926 | 1.437941 | 1.238945 | 1.345937451 | 0.026917 |
| py04293.t1 | py04293.t1 | 1.433187 | 1.379842 | 1.646603 | 1.486543995 | 0.026929 |
| py07318.t1 | py07318.t1 | 1.264475 | 1.228607 | 1.391549 | 1.29487689 | 0.026971 |
| py08379.t1 | py08379.t1 | 1.538446 | 1.551193 | 1.309144 | 1.46626093 | 0.027291 |
| py03713.t1 | py03713.t1 | 1.818585 | 1.592004 | 1.457115 | 1.622568136 | 0.027516 |
| py10776.t1 | py10776.t1 | 2.157136 | 2.696451 | 1.975396 | 2.276327869 | 0.027592 |
| py02951.t1 | py02951.t1 | 1.616767 | 1.943847 | 1.557239 | 1.705950795 | 0.027781 |
| py08655.t1 | py08655.t1 | 1.398621 | 1.521197 | 1.28438 | 1.401399493 | 0.027813 |
| py04865.t1 | py04865.t1 | 1.261428 | 1.231158 | 1.141129 | 1.211238162 | 0.028026 |
| py08217.t1 | py08217.t1 | 1.68135 | 2.18347 | 1.795428 | 1.886749317 | 0.028138 |
| py06045.t1 | py06045.t1 | 1.956082 | 2.149519 | 1.616642 | 1.907414384 | 0.028217 |
| py08904.t1 | py08904.t1 | 1.23849 | 1.254877 | 1.399435 | 1.297600814 | 0.028279 |
| py10461.t1 | py10461.t1 | 1.245601 | 1.34559 | 1.192403 | 1.261197887 | 0.028306 |
| py01287.t1 | py01287.t1 | 1.328564 | 1.322192 | 1.181815 | 1.277523402 | 0.02851 |
| py09399.t1 | py09399.t1 | 1.186295 | 1.346263 | 1.312879 | 1.281812312 | 0.028613 |
| py05205.t1 | py05205.t1 | 1.258421 | 1.250286 | 1.141353 | 1.216686526 | 0.029021 |
| py10835.t1 | py10835.t1 | 1.286072 | 1.288328 | 1.159343 | 1.244581286 | 0.029054 |
| py03110.t1 | py03110.t1 | 1.244075 | 1.294544 | 1.155512 | 1.231376784 | 0.029485 |
| py05695.t1 | py05695.t1 | 1.170241 | 1.24077 | 1.318771 | 1.243260597 | 0.029715 |
| py11231.t1 | py11231.t1 | 1.201107 | 1.312307 | 1.18095 | 1.231454712 | 0.029756 |
| py05276.t1 | py05276.t1 | 1.218934 | 1.255262 | 1.133745 | 1.202646797 | 0.030159 |
| py09427.t1 | py09427.t1 | 1.38309 | 1.250499 | 1.478106 | 1.37056517 | 0.03029 |
| py08828.t1 | py08828.t1 | 1.394728 | 1.420184 | 1.223636 | 1.3461827 | 0.03034 |
| py06977.t6 | py06977.t6 | 1.495943 | 1.825263 | 1.500498 | 1.607234338 | 0.030755 |
| py02335.t1 | py02335.t1 | 1.254667 | 1.409102 | 1.239775 | 1.301181475 | 0.030817 |
| py06677.t1 | py06677.t1 | 1.494075 | 1.441888 | 1.258138 | 1.398033657 | 0.030828 |
| py02488.t1 | py02488.t1 | 1.278584 | 1.232225 | 1.14446 | 1.218422935 | 0.030924 |
| py00877.t1 | py00877.t1 | 1.200797 | 1.287761 | 1.155827 | 1.214795154 | 0.030999 |
| py09017.t1 | py09017.t1 | 1.269367 | 1.351589 | 1.501774 | 1.374243455 | 0.0315 |
| py07137.t1 | py07137.t1 | 1.534428 | 1.478491 | 1.276848 | 1.429922505 | 0.031546 |
| py02428.t1 | py02428.t1 | 1.283104 | 1.200565 | 1.150451 | 1.211373274 | 0.031882 |
| py03416.t1 | py03416.t1 | 1.360323 | 1.584461 | 1.339341 | 1.428041609 | 0.031983 |
| py09105.t1 | py09105.t1 | 1.204438 | 1.301657 | 1.163677 | 1.223257385 | 0.032002 |
| py06421.t1 | py06421.t1 | 1.328561 | 1.400065 | 1.204078 | 1.310901492 | 0.032288 |
| py09600.t1 | py09600.t1 | 1.186483 | 1.28074 | 1.152814 | 1.20667891 | 0.032643 |
| py00247.t1 | py00247.t1 | 1.510057 | 1.333795 | 1.655135 | 1.499662392 | 0.032879 |
| py04877.t1 | py04877.t1 | 1.457266 | 1.237663 | 1.452241 | 1.382390331 | 0.034009 |
| py07881.t1 | py07881.t1 | 1.644926 | 2.246322 | 1.87437 | 1.921872541 | 0.034282 |
| py09825.t1 | py09825.t1 | 1.581797 | 1.391505 | 1.777668 | 1.583656743 | 0.034599 |
| py06282.t1 | py06282.t1 | 1.16269 | 1.278933 | 1.163853 | 1.201825328 | 0.03461 |
| py01724.t1 | py01724.t1 | 1.204286 | 1.269259 | 1.134803 | 1.202782804 | 0.034751 |
| py00954.t1 | py00954.t1 | 1.171975 | 1.318985 | 1.20457 | 1.231843212 | 0.035034 |
| py07399.t1 | py07399.t1 | 1.424385 | 1.586604 | 1.294524 | 1.435170667 | 0.035689 |
| py00034.t1 | py00034.t1 | 1.220949 | 1.159848 | 1.314867 | 1.231887817 | 0.035782 |
| py00879.t1 | py00879.t1 | 1.172685 | 1.161096 | 1.288585 | 1.207455482 | 0.036405 |
| py08635.t1 | py08635.t1 | 1.210578 | 1.303547 | 1.153479 | 1.222534792 | 0.036516 |
| py00827.t1 | py00827.t1 | 1.311069 | 1.37368 | 1.180995 | 1.28858138 | 0.036562 |
| py07013.t1 | py07013.t1 | 1.407404 | 1.1972 | 1.333425 | 1.31267611 | 0.036646 |
| py05388.t1 | py05388.t1 | 1.148049 | 1.266789 | 1.305737 | 1.240191643 | 0.036842 |
| py06715.t1 | py06715.t1 | 1.296084 | 1.204542 | 1.149356 | 1.216660539 | 0.036859 |
| py05578.t1 | py05578.t1 | 1.277621 | 1.34885 | 1.168754 | 1.265074964 | 0.036882 |
| py00331.t1 | py00331.t1 | 1.147445 | 1.199707 | 1.291447 | 1.212866117 | 0.03694 |
| py05643.t1 | py05643.t1 | 1.257731 | 1.352376 | 1.511309 | 1.373805266 | 0.03701 |
| py02637.t1 | py02637.t1 | 1.217686 | 1.310013 | 1.154745 | 1.227481149 | 0.037113 |
| py04024.t1 | py04024.t1 | 1.444629 | 1.44907 | 1.224365 | 1.372688291 | 0.037401 |
| py02128.t1 | py02128.t1 | 1.39088 | 1.400256 | 1.197802 | 1.329645874 | 0.037802 |
| py06384.t1 | py06384.t1 | 1.282547 | 1.175364 | 1.149572 | 1.202494331 | 0.038127 |
| py03290.t1 | py03290.t1 | 1.216495 | 1.330906 | 1.169 | 1.238800277 | 0.038184 |
| py00957.t1 | py00957.t1 | 1.326089 | 1.413494 | 1.195925 | 1.31183602 | 0.038718 |
| py11176.t1 | py11176.t1 | 1.290807 | 1.371694 | 1.176201 | 1.27956713 | 0.038775 |
| py03586.t2 | py03586.t2 | 1.262737 | 1.27078 | 1.13186 | 1.221792223 | 0.038829 |
| py02914.t1 | py02914.t1 | 1.469078 | 1.320054 | 1.658771 | 1.482634247 | 0.038854 |
| py01286.t1 | py01286.t1 | 1.278936 | 1.272401 | 1.134005 | 1.228447352 | 0.04023 |
| py08744.t1 | py08744.t1 | 1.248206 | 1.164232 | 1.345132 | 1.252523333 | 0.040269 |
| py07449.t1 | py07449.t1 | 1.162311 | 1.156588 | 1.285482 | 1.201460525 | 0.040899 |
| py07279.t1 | py07279.t1 | 2.046404 | 2.111572 | 2.930463 | 2.362812983 | 0.04091 |
| py00774.t1 | py00774.t1 | 1.160313 | 1.240709 | 1.341512 | 1.247511306 | 0.042043 |
| py03790.t1 | py03790.t1 | 1.389726 | 1.246654 | 1.193264 | 1.276547786 | 0.042155 |
| py07431.t1 | py07431.t1 | 1.278544 | 1.232357 | 1.125207 | 1.212036022 | 0.042944 |
| py04703.t1 | py04703.t1 | 1.456649 | 1.227843 | 1.281523 | 1.322004974 | 0.043075 |
| py03329.t1 | py03329.t1 | 1.17762 | 1.294208 | 1.147499 | 1.206442423 | 0.04389 |
| py04023.t1 | py04023.t1 | 1.349507 | 1.388799 | 1.677414 | 1.47190651 | 0.04479 |
| py10172.t1 | py10172.t1 | 1.151648 | 1.20416 | 1.319476 | 1.225094851 | 0.045224 |
| py04155.t1 | py04155.t1 | 1.2588 | 1.387659 | 1.178636 | 1.275031564 | 0.045673 |
| py08983.t1 | py08983.t1 | 1.230939 | 1.422996 | 1.225254 | 1.293063037 | 0.04582 |
| py07276.t1 | py07276.t1 | 1.392353 | 1.698527 | 1.357395 | 1.482758331 | 0.046865 |
| py11089.t1 | py11089.t1 | 1.262897 | 1.541511 | 1.323664 | 1.376023996 | 0.047055 |
| py10984.t1 | py10984.t1 | 1.431606 | 1.484156 | 1.208772 | 1.374844764 | 0.047152 |
| py08381.t1 | py08381.t1 | 1.146008 | 1.331209 | 1.324736 | 1.267317892 | 0.047863 |
| py02367.t1 | py02367.t1 | 1.738758 | 2.35332 | 1.695868 | 1.929315196 | 0.048456 |
| py08543.t1 | py08543.t1 | 1.399355 | 1.438394 | 1.186701 | 1.341483262 | 0.048655 |
| py11334.t1 | py11334.t1 | 0.669364 | 0.668861 | 0.661547 | 0.666590802 | 5.74E-05 |
| py10772.t1 | py10772.t1 | 0.732713 | 0.736604 | 0.727583 | 0.732299893 | 9.52E-05 |
| py08158.t1 | py08158.t1 | 0.767058 | 0.773576 | 0.765162 | 0.768598597 | 0.000121 |
| py09507.t1 | py09507.t1 | 0.647394 | 0.64089 | 0.657793 | 0.64869245 | 0.000196 |
| py05645.t1 | py05645.t1 | 0.477272 | 0.498984 | 0.498862 | 0.49170603 | 0.000202 |
| py04444.t1 | py04444.t1 | 0.525775 | 0.535977 | 0.548699 | 0.536817077 | 0.000205 |
| py01355.t1 | py01355.t1 | 0.635956 | 0.622331 | 0.642751 | 0.633679358 | 0.000269 |
| py10505.t1 | py10505.t1 | 0.569516 | 0.554299 | 0.543744 | 0.555852911 | 0.000284 |
| py05061.t1 | py05061.t1 | 0.727118 | 0.743324 | 0.735128 | 0.735189666 | 0.000312 |
| py09428.t1 | py09428.t1 | 0.673815 | 0.683587 | 0.695038 | 0.684146606 | 0.000377 |
| py09111.t1 | py09111.t1 | 0.68672 | 0.706988 | 0.692973 | 0.69556018 | 0.000387 |
| py02584.t1 | py02584.t1 | 0.811022 | 0.799445 | 0.797972 | 0.802813048 | 0.000438 |
| py07316.t1 | py07316.t1 | 0.512092 | 0.503521 | 0.475922 | 0.497178435 | 0.000471 |
| py06897.t1 | py06897.t1 | 0.224313 | 0.166707 | 0.210557 | 0.200525699 | 0.000472 |
| py05683.t1 | py05683.t1 | 0.748734 | 0.728657 | 0.734202 | 0.737197528 | 0.000518 |
| py10417.t1 | py10417.t1 | 0.715206 | 0.708904 | 0.730556 | 0.718222172 | 0.00052 |
| py05293.t1 | py05293.t1 | 0.536136 | 0.526684 | 0.499264 | 0.520694465 | 0.000532 |
| py02770.t1 | py02770.t1 | 0.567505 | 0.532279 | 0.560652 | 0.553478742 | 0.000583 |
| py08867.t1 | py08867.t1 | 0.647496 | 0.617484 | 0.62153 | 0.628836716 | 0.000641 |
| py00627.t1 | py00627.t1 | 0.645875 | 0.614013 | 0.622539 | 0.627475626 | 0.000653 |
| py08253.t1 | py08253.t1 | 0.766105 | 0.783434 | 0.765684 | 0.771740732 | 0.000656 |
| py11527.t1 | py11527.t1 | 0.753836 | 0.753061 | 0.772276 | 0.759724277 | 0.000682 |
| py01131.t1 | py01131.t1 | 0.79615 | 0.79448 | 0.777609 | 0.789412837 | 0.00079 |
| py00862.t1 | py00862.t1 | 0.19856 | 0.124486 | 0.128888 | 0.150644456 | 0.000797 |
| py00158.t1 | py00158.t1 | 0.647701 | 0.630827 | 0.665378 | 0.647968898 | 0.000802 |
| py00589.t1 | py00589.t1 | 0.501926 | 0.497323 | 0.541536 | 0.513595049 | 0.000831 |
| py10831.t1 | py10831.t1 | 0.798655 | 0.787853 | 0.810226 | 0.798911377 | 0.00103 |
| py02015.t1 | py02015.t1 | 0.733356 | 0.705147 | 0.730506 | 0.723002963 | 0.001046 |
| py02339.t1 | py02339.t1 | 0.578342 | 0.617583 | 0.574741 | 0.590222028 | 0.001119 |
| py07231.t1 | py07231.t1 | 0.82614 | 0.810433 | 0.804984 | 0.813852141 | 0.001159 |
| py05907.t1 | py05907.t1 | 0.648936 | 0.667912 | 0.688736 | 0.668527934 | 0.0012 |
| py00423.t1 | py00423.t1 | 0.561344 | 0.52274 | 0.57498 | 0.553021481 | 0.001223 |
| py00118.t1 | py00118.t1 | 0.611539 | 0.561765 | 0.570698 | 0.581333996 | 0.001337 |
| py11211.t1 | py11211.t1 | 0.720471 | 0.711117 | 0.684455 | 0.705347479 | 0.001338 |
| py07533.t1 | py07533.t1 | 0.764802 | 0.76321 | 0.736923 | 0.754978374 | 0.001358 |
| py03665.t1 | py03665.t1 | 0.622426 | 0.569878 | 0.59024 | 0.59418162 | 0.001418 |
| py09896.t1 | py09896.t1 | 0.693907 | 0.677793 | 0.717424 | 0.696375033 | 0.001433 |
| py10773.t1 | py10773.t1 | 0.551383 | 0.504874 | 0.56188 | 0.539379288 | 0.001443 |
| py05565.t1 | py05565.t1 | 0.781846 | 0.78988 | 0.761443 | 0.777722998 | 0.001447 |
| py02233.t1 | py02233.t1 | 0.708745 | 0.706391 | 0.740178 | 0.718437978 | 0.001493 |
| py09973.t1 | py09973.t1 | 0.702779 | 0.726042 | 0.740597 | 0.723139179 | 0.001579 |
| py01519.t1 | py01519.t1 | 0.83903 | 0.816458 | 0.835956 | 0.830481188 | 0.001734 |
| py05539.t1 | py05539.t1 | 0.652829 | 0.603031 | 0.607154 | 0.621004481 | 0.001768 |
| py08916.t1 | py08916.t1 | 0.807639 | 0.782938 | 0.80908 | 0.799885495 | 0.001793 |
| py05516.t1 | py05516.t1 | 0.824207 | 0.82159 | 0.799427 | 0.815074609 | 0.001802 |
| py09348.t1 | py09348.t1 | 0.73877 | 0.698195 | 0.724574 | 0.720512845 | 0.001804 |
| py03809.t1 | py03809.t1 | 0.764445 | 0.734988 | 0.728758 | 0.742730531 | 0.001825 |
| py02140.t1 | py02140.t1 | 0.798277 | 0.765956 | 0.78054 | 0.781591246 | 0.001826 |
| py00599.t2 | py00599.t2 | 0.551794 | 0.49035 | 0.549545 | 0.53056312 | 0.001831 |
| py10492.t1 | py10492.t1 | 0.757396 | 0.785963 | 0.787124 | 0.776827763 | 0.001892 |
| py09680.t1 | py09680.t1 | 0.562911 | 0.602004 | 0.624014 | 0.596309827 | 0.001953 |
| py07480.t1 | py07480.t1 | 0.753952 | 0.781044 | 0.745279 | 0.760091531 | 0.00201 |
| py04647.t1 | py04647.t1 | 0.650686 | 0.602811 | 0.653078 | 0.63552495 | 0.002012 |
| py11103.t1 | py11103.t1 | 0.70541 | 0.730512 | 0.747523 | 0.727814872 | 0.002013 |
| py08554.t1 | py08554.t1 | 0.668644 | 0.613879 | 0.651131 | 0.644551139 | 0.002058 |
| py07766.t1 | py07766.t1 | 0.583928 | 0.533117 | 0.598444 | 0.571829642 | 0.002133 |
| py08358.t1 | py08358.t1 | 0.722947 | 0.750253 | 0.76374 | 0.745646736 | 0.002218 |
| py03434.t1 | py03434.t1 | 0.768121 | 0.727731 | 0.754948 | 0.750266588 | 0.00226 |
| py07471.t1 | py07471.t1 | 0.717442 | 0.675017 | 0.717409 | 0.703289197 | 0.002262 |
| py04685.t1 | py04685.t1 | 0.778894 | 0.757339 | 0.738884 | 0.758372138 | 0.002282 |
| pyi00476.t2 | pyi00476.t2 | 0.770037 | 0.798897 | 0.764567 | 0.77783355 | 0.00229 |
| py04195.t1 | py04195.t1 | 0.558998 | 0.626861 | 0.587787 | 0.591215406 | 0.002306 |
| py02877.t1 | py02877.t1 | 0.630265 | 0.616306 | 0.673798 | 0.640122955 | 0.002306 |
| py04420.t1 | py04420.t1 | 0.78344 | 0.812689 | 0.812104 | 0.80274442 | 0.002387 |
| py09970.t1 | py09970.t1 | 0.648787 | 0.599333 | 0.656997 | 0.635039204 | 0.002426 |
| py08588.t1 | py08588.t1 | 0.826245 | 0.807067 | 0.793622 | 0.808978028 | 0.002446 |
| py09848.t1 | py09848.t1 | 0.838181 | 0.830437 | 0.809291 | 0.825969684 | 0.002452 |
| py10313.t1 | py10313.t1 | 0.638197 | 0.690289 | 0.683847 | 0.670777555 | 0.002471 |
| py10954.t1 | py10954.t1 | 0.421583 | 0.452914 | 0.513273 | 0.462590165 | 0.002497 |
| py09981.t1 | py09981.t1 | 0.70597 | 0.650633 | 0.68384 | 0.680147394 | 0.002518 |
| py10755.t1 | py10755.t1 | 0.690425 | 0.673676 | 0.63335 | 0.665817402 | 0.002559 |
| py00438.t1 | py00438.t1 | 0.816621 | 0.816287 | 0.786971 | 0.80662643 | 0.002573 |
| py02223.t1 | py02223.t1 | 0.787307 | 0.815968 | 0.782624 | 0.795299903 | 0.002582 |
| py04634.t1 | py04634.t1 | 0.769329 | 0.784247 | 0.742778 | 0.765451015 | 0.002663 |
| py05510.t1 | py05510.t1 | 0.722123 | 0.751265 | 0.767039 | 0.746809121 | 0.002689 |
| py01876.t1 | py01876.t1 | 0.713145 | 0.761 | 0.73797 | 0.737371697 | 0.002757 |
| py10495.t1 | py10495.t1 | 0.729516 | 0.77627 | 0.748257 | 0.751347691 | 0.002972 |
| py00874.t1 | py00874.t1 | 0.675797 | 0.671352 | 0.72424 | 0.690463162 | 0.002981 |
| py06387.t1 | py06387.t1 | 0.805543 | 0.805826 | 0.771784 | 0.794384201 | 0.003007 |
| py03379.t2 | py03379.t2 | 0.614111 | 0.657868 | 0.584971 | 0.618983093 | 0.003077 |
| py10368.t1 | py10368.t1 | 0.792618 | 0.748601 | 0.764197 | 0.768471958 | 0.003083 |
| py03315.t1 | py03315.t1 | 0.560256 | 0.626372 | 0.552516 | 0.579714438 | 0.003095 |
| py01940.t1 | py01940.t1 | 0.765703 | 0.720323 | 0.722848 | 0.736291514 | 0.003103 |
| py06270.t1 | py06270.t1 | 0.818874 | 0.834208 | 0.798284 | 0.817121586 | 0.003223 |
| py06976.t1 | py06976.t1 | 0.81674 | 0.790731 | 0.826951 | 0.811473843 | 0.003255 |
| py11338.t1 | py11338.t1 | 0.787443 | 0.756988 | 0.799135 | 0.781188896 | 0.00328 |
| py05925.t1 | py05925.t1 | 0.776039 | 0.789372 | 0.816305 | 0.793905354 | 0.003286 |
| py03151.t1 | py03151.t1 | 0.81622 | 0.781985 | 0.778741 | 0.792315141 | 0.003316 |
| py11233.t1 | py11233.t1 | 0.811068 | 0.769896 | 0.780678 | 0.787213794 | 0.003339 |
| py10873.t1 | py10873.t1 | 0.790966 | 0.809731 | 0.829331 | 0.810009124 | 0.003381 |
| py11309.t1 | py11309.t1 | 0.646994 | 0.649721 | 0.706491 | 0.667735345 | 0.00339 |
| py00493.t1 | py00493.t1 | 0.393427 | 0.451026 | 0.504677 | 0.449710018 | 0.00339 |
| py01593.t1 | py01593.t1 | 0.499163 | 0.454802 | 0.55521 | 0.503058122 | 0.0034 |
| py08917.t1 | py08917.t1 | 0.550689 | 0.630593 | 0.5648 | 0.582027477 | 0.003452 |
| py11003.t1 | py11003.t1 | 0.820768 | 0.788608 | 0.823186 | 0.810854243 | 0.003454 |
| py09091.t1 | py09091.t1 | 0.651389 | 0.625294 | 0.695275 | 0.65731958 | 0.003531 |
| py03767.t1 | py03767.t1 | 0.76368 | 0.714308 | 0.756701 | 0.74489656 | 0.003637 |
| py06493.t1 | py06493.t1 | 0.850961 | 0.830813 | 0.815986 | 0.832586951 | 0.003645 |
| py07674.t1 | py07674.t1 | 0.809551 | 0.788412 | 0.764359 | 0.787440461 | 0.003751 |
| py02110.t1 | py02110.t1 | 0.840716 | 0.804602 | 0.810895 | 0.818737595 | 0.003755 |
| py07915.t1 | py07915.t1 | 0.740477 | 0.785884 | 0.740905 | 0.755755236 | 0.003783 |
| py06383.t1 | py06383.t1 | 0.621201 | 0.638241 | 0.693004 | 0.650815222 | 0.003826 |
| py05765.t1 | py05765.t1 | 0.844642 | 0.836735 | 0.809584 | 0.830319949 | 0.003892 |
| py08849.t1 | py08849.t1 | 0.815594 | 0.849909 | 0.819031 | 0.828178239 | 0.004008 |
| py09397.t1 | py09397.t1 | 0.76117 | 0.808798 | 0.783241 | 0.78440271 | 0.004049 |
| py02116.t1 | py02116.t1 | 0.724851 | 0.690755 | 0.656449 | 0.690684912 | 0.004051 |
| py01054.t1 | py01054.t1 | 0.750215 | 0.693805 | 0.740496 | 0.728172031 | 0.004077 |
| py02474.t1 | py02474.t1 | 0.792934 | 0.749192 | 0.745355 | 0.76249359 | 0.004103 |
| py07687.t1 | py07687.t1 | 0.7069 | 0.678699 | 0.743422 | 0.709673634 | 0.004139 |
| py01440.t1 | py01440.t1 | 0.850962 | 0.813174 | 0.828316 | 0.830817404 | 0.004186 |
| py05936.t1 | py05936.t1 | 0.82344 | 0.785723 | 0.782229 | 0.797130709 | 0.004203 |
| py09759.t1 | py09759.t1 | 0.721529 | 0.65318 | 0.700642 | 0.691783504 | 0.004277 |
| py08546.t1 | py08546.t1 | 0.63607 | 0.611719 | 0.690539 | 0.646109517 | 0.004307 |
| py02600.t1 | py02600.t1 | 0.65771 | 0.61408 | 0.569393 | 0.613727699 | 0.004328 |
| py00552.t1 | py00552.t1 | 0.776845 | 0.728333 | 0.775649 | 0.760276012 | 0.004411 |
| py02852.t1 | py02852.t1 | 0.804392 | 0.838385 | 0.798055 | 0.813610762 | 0.004483 |
| py06363.t1 | py06363.t1 | 0.573377 | 0.653523 | 0.644178 | 0.623692729 | 0.004491 |
| py10154.t1 | py10154.t1 | 0.664187 | 0.703709 | 0.734054 | 0.700649875 | 0.004534 |
| py06104.t1 | py06104.t1 | 0.672138 | 0.584891 | 0.623855 | 0.626961427 | 0.004545 |
| py02023.t1 | py02023.t1 | 0.787656 | 0.764201 | 0.731856 | 0.761237587 | 0.004559 |
| py10730.t1 | py10730.t1 | 0.759863 | 0.699085 | 0.709205 | 0.722717307 | 0.004566 |
| py08449.t1 | py08449.t1 | 0.691389 | 0.668278 | 0.737798 | 0.699155169 | 0.004585 |
| py03652.t1 | py03652.t1 | 0.653532 | 0.675211 | 0.725846 | 0.684862903 | 0.004591 |
| py05588.t1 | py05588.t1 | 0.611025 | 0.640798 | 0.548521 | 0.600114967 | 0.004592 |
| py04843.t1 | py04843.t1 | 0.653324 | 0.693527 | 0.726239 | 0.691029839 | 0.004625 |
| py02578.t1 | py02578.t1 | 0.785445 | 0.762071 | 0.728501 | 0.758672233 | 0.004657 |
| py04054.t1 | py04054.t1 | 0.670497 | 0.651684 | 0.724582 | 0.682254456 | 0.004695 |
| py04414.t1 | py04414.t1 | 0.675913 | 0.589525 | 0.613926 | 0.626454747 | 0.004705 |
| py01580.t1 | py01580.t1 | 0.73877 | 0.672385 | 0.680605 | 0.697253325 | 0.004729 |
| py04973.t1 | py04973.t1 | 0.700356 | 0.687517 | 0.626809 | 0.671560744 | 0.004735 |
| py04523.t1 | py04523.t1 | 0.773841 | 0.712512 | 0.730773 | 0.7390418 | 0.004819 |
| py10124.t1 | py10124.t1 | 0.820005 | 0.849381 | 0.80854 | 0.825975501 | 0.004849 |
| py08500.t1 | py08500.t1 | 0.700319 | 0.763862 | 0.71586 | 0.726680368 | 0.00486 |
| py06862.t1 | py06862.t1 | 0.777917 | 0.791876 | 0.825758 | 0.798516991 | 0.004933 |
| py01043.t1 | py01043.t1 | 0.796492 | 0.832289 | 0.836284 | 0.821688114 | 0.004996 |
| py07397.t1 | py07397.t1 | 0.579192 | 0.664434 | 0.580816 | 0.608147071 | 0.00512 |
| py05441.t1 | py05441.t1 | 0.750241 | 0.78456 | 0.804656 | 0.779819163 | 0.005165 |
| py11259.t1 | py11259.t1 | 0.808376 | 0.82039 | 0.850557 | 0.826440724 | 0.005185 |
| py08261.t1 | py08261.t1 | 0.629338 | 0.626528 | 0.538632 | 0.598165925 | 0.005447 |
| py04246.t1 | py04246.t1 | 0.83342 | 0.784423 | 0.810362 | 0.809401557 | 0.005468 |
| py03214.t1 | py03214.t1 | 0.750037 | 0.675913 | 0.705699 | 0.710549958 | 0.00549 |
| py08208.t1 | py08208.t1 | 0.682089 | 0.588683 | 0.641449 | 0.637407 | 0.005515 |
| py02208.t1 | py02208.t1 | 0.70327 | 0.644316 | 0.722282 | 0.689956078 | 0.005682 |
| py07741.t1 | py07741.t1 | 0.823161 | 0.774091 | 0.812782 | 0.803344484 | 0.005715 |
| py04129.t1 | py04129.t1 | 0.717785 | 0.689993 | 0.762576 | 0.723451091 | 0.005795 |
| py05853.t1 | py05853.t1 | 0.774367 | 0.77025 | 0.820784 | 0.788467097 | 0.005815 |
| py00817.t1 | py00817.t1 | 0.749243 | 0.728594 | 0.792211 | 0.756682379 | 0.005878 |
| py02759.t1 | py02759.t1 | 0.807769 | 0.841496 | 0.84993 | 0.833065203 | 0.005901 |
| py04223.t1 | py04223.t1 | 0.708919 | 0.776119 | 0.72243 | 0.735822482 | 0.005981 |
| py05226.t1 | py05226.t1 | 0.75174 | 0.713162 | 0.78067 | 0.748523959 | 0.005992 |
| py11136.t1 | py11136.t1 | 0.729151 | 0.66597 | 0.737141 | 0.710753781 | 0.006002 |
| py10402.t1 | py10402.t1 | 0.768911 | 0.821662 | 0.80649 | 0.79902079 | 0.006031 |
| py04642.t1 | py04642.t1 | 0.743838 | 0.678148 | 0.743094 | 0.721693462 | 0.006065 |
| py04113.t1 | py04113.t1 | 0.656464 | 0.548052 | 0.591631 | 0.598715582 | 0.006104 |
| py02113.t1 | py02113.t1 | 0.73093 | 0.645514 | 0.680317 | 0.685586895 | 0.006163 |
| py01216.t1 | py01216.t1 | 0.748432 | 0.700762 | 0.667722 | 0.705638435 | 0.006274 |
| py07654.t1 | py07654.t1 | 0.702884 | 0.628666 | 0.610046 | 0.647198712 | 0.006398 |
| py03068.t1 | py03068.t1 | 0.807896 | 0.754322 | 0.802452 | 0.788223118 | 0.0064 |
| py01960.t1 | py01960.t1 | 0.77347 | 0.74756 | 0.701393 | 0.740807599 | 0.006549 |
| py05608.t1 | py05608.t1 | 0.541539 | 0.444709 | 0.574804 | 0.520350791 | 0.006553 |
| py00497.t1 | py00497.t1 | 0.836042 | 0.786762 | 0.789677 | 0.804160139 | 0.006578 |
| py04563.t1 | py04563.t1 | 0.689079 | 0.673008 | 0.753332 | 0.70513943 | 0.006855 |
| py05301.t1 | py05301.t1 | 0.444794 | 0.570867 | 0.437706 | 0.484455411 | 0.006966 |
| py09682.t1 | py09682.t1 | 0.817952 | 0.760342 | 0.763941 | 0.780744874 | 0.007145 |
| py08794.t1 | py08794.t1 | 0.73141 | 0.717611 | 0.78856 | 0.745860471 | 0.007224 |
| py08538.t1 | py08538.t1 | 0.680772 | 0.763506 | 0.721163 | 0.721813429 | 0.007292 |
| py09659.t1 | py09659.t1 | 0.74962 | 0.812051 | 0.758829 | 0.773499949 | 0.007299 |
| py09131.t1 | py09131.t1 | 0.839035 | 0.781418 | 0.804385 | 0.808279499 | 0.007543 |
| py08628.t1 | py08628.t1 | 0.723257 | 0.671749 | 0.623975 | 0.672993795 | 0.007598 |
| py00244.t1 | py00244.t1 | 0.523343 | 0.483328 | 0.619031 | 0.541900753 | 0.007635 |
| py00782.t1 | py00782.t1 | 0.679868 | 0.564988 | 0.603337 | 0.616064299 | 0.007647 |
| py01655.t1 | py01655.t1 | 0.808911 | 0.819266 | 0.76042 | 0.796199136 | 0.007827 |
| py04173.t1 | py04173.t1 | 0.834601 | 0.833577 | 0.785126 | 0.817768387 | 0.007929 |
| py05776.t1 | py05776.t1 | 0.790256 | 0.766469 | 0.829982 | 0.795569151 | 0.008113 |
| py06064.t1 | py06064.t1 | 0.734704 | 0.759417 | 0.807011 | 0.767044033 | 0.008195 |
| py04303.t1 | py04303.t1 | 0.783304 | 0.767259 | 0.708629 | 0.753063959 | 0.008341 |
| py11492.t1 | py11492.t1 | 0.798722 | 0.841986 | 0.848842 | 0.829850052 | 0.008395 |
| py06416.t1 | py06416.t1 | 0.666285 | 0.635285 | 0.547996 | 0.616521935 | 0.00842 |
| py08715.t1 | py08715.t1 | 0.776749 | 0.787146 | 0.836651 | 0.800182073 | 0.008445 |
| py05280.t1 | py05280.t1 | 0.636061 | 0.664294 | 0.73715 | 0.679168354 | 0.008696 |
| py04353.t1 | py04353.t1 | 0.767106 | 0.67702 | 0.72555 | 0.723225261 | 0.00873 |
| py04706.t1 | py04706.t1 | 0.720762 | 0.695706 | 0.620214 | 0.678894039 | 0.00874 |
| py07468.t1 | py07468.t1 | 0.8141 | 0.8102 | 0.860688 | 0.82832944 | 0.008808 |
| py09231.t1 | py09231.t1 | 0.740577 | 0.784251 | 0.698747 | 0.741191635 | 0.008975 |
| py04592.t1 | py04592.t1 | 0.703243 | 0.741964 | 0.78776 | 0.744322173 | 0.009004 |
| py05514.t1 | py05514.t1 | 0.787014 | 0.701768 | 0.737828 | 0.742203438 | 0.009059 |
| py02195.t1 | py02195.t1 | 0.685545 | 0.648926 | 0.749356 | 0.694608902 | 0.009107 |
| py10226.t1 | py10226.t1 | 0.777907 | 0.697438 | 0.761488 | 0.745610887 | 0.009184 |
| py00697.t1 | py00697.t1 | 0.702672 | 0.60251 | 0.589604 | 0.631595327 | 0.009277 |
| py02949.t1 | py02949.t1 | 0.716272 | 0.794539 | 0.771499 | 0.760769847 | 0.009291 |
| py00618.t1 | py00618.t1 | 0.828389 | 0.764672 | 0.767097 | 0.786719312 | 0.009419 |
| py04125.t1 | py04125.t1 | 0.769188 | 0.76239 | 0.82822 | 0.786599263 | 0.009459 |
| py08602.t1 | py08602.t1 | 0.835171 | 0.797297 | 0.854598 | 0.829021927 | 0.009545 |
| py10520.t1 | py10520.t1 | 0.724758 | 0.681776 | 0.775074 | 0.727202699 | 0.009627 |
| py04142.t1 | py04142.t1 | 0.820298 | 0.855036 | 0.794534 | 0.823289376 | 0.009697 |
| py10448.t1 | py10448.t1 | 0.717717 | 0.799481 | 0.736954 | 0.751383669 | 0.009712 |
| py04070.t1 | py04070.t1 | 0.758447 | 0.723998 | 0.661597 | 0.714680439 | 0.009725 |
| py10842.t1 | py10842.t1 | 0.75653 | 0.730259 | 0.661449 | 0.716079335 | 0.009823 |
| py05442.t1 | py05442.t1 | 0.655748 | 0.56772 | 0.68911 | 0.637525956 | 0.009831 |
| py05452.t1 | py05452.t1 | 0.755334 | 0.816219 | 0.817107 | 0.79621991 | 0.009916 |
| py09225.t1 | py09225.t1 | 0.718049 | 0.717547 | 0.795093 | 0.743563136 | 0.009945 |
| py04552.t1 | py04552.t1 | 0.724478 | 0.658145 | 0.757462 | 0.713361781 | 0.010222 |
| py08996.t1 | py08996.t1 | 0.744142 | 0.764109 | 0.82042 | 0.776223631 | 0.010255 |
| py02219.t1 | py02219.t1 | 0.61125 | 0.560988 | 0.69363 | 0.621956086 | 0.010298 |
| py00526.t1 | py00526.t1 | 0.807079 | 0.735618 | 0.798559 | 0.780418998 | 0.010369 |
| py05893.t1 | py05893.t1 | 0.794833 | 0.746187 | 0.705326 | 0.748782007 | 0.01044 |
| py06128.t1 | py06128.t1 | 0.505405 | 0.619995 | 0.642505 | 0.589301389 | 0.010514 |
| py00319.t1 | py00319.t1 | 0.77997 | 0.76798 | 0.837737 | 0.795229005 | 0.010879 |
| py05174.t1 | py05174.t1 | 0.633421 | 0.729388 | 0.728682 | 0.697163954 | 0.010896 |
| py05080.t1 | py05080.t1 | 0.797841 | 0.775138 | 0.844651 | 0.805876383 | 0.010932 |
| py10771.t1 | py10771.t1 | 0.782345 | 0.705002 | 0.688268 | 0.72520493 | 0.010936 |
| py11212.t1 | py11212.t1 | 0.721842 | 0.658178 | 0.76274 | 0.714253417 | 0.011146 |
| py05656.t1 | py05656.t1 | 0.748258 | 0.749219 | 0.821291 | 0.772922665 | 0.011155 |
| py08219.t1 | py08219.t1 | 0.54603 | 0.518261 | 0.665785 | 0.57669189 | 0.011241 |
| py08498.t1 | py08498.t1 | 0.827231 | 0.748025 | 0.779454 | 0.784903655 | 0.011267 |
| py03703.t1 | py03703.t1 | 0.835547 | 0.760109 | 0.797984 | 0.797879967 | 0.01141 |
| py04172.t1 | py04172.t1 | 0.661388 | 0.727965 | 0.765723 | 0.718358633 | 0.011525 |
| py08621.t1 | py08621.t1 | 0.750542 | 0.809938 | 0.823167 | 0.794549096 | 0.01161 |
| py01749.t1 | py01749.t1 | 0.676597 | 0.639107 | 0.753764 | 0.689822425 | 0.011635 |
| py00705.t1 | py00705.t1 | 0.65706 | 0.753384 | 0.745991 | 0.718811444 | 0.011899 |
| py05268.t1 | py05268.t1 | 0.834499 | 0.766094 | 0.823134 | 0.807909104 | 0.011921 |
| py10833.t1 | py10833.t1 | 0.739052 | 0.690347 | 0.790424 | 0.739940801 | 0.01212 |
| py08361.t1 | py08361.t1 | 0.762662 | 0.649004 | 0.703023 | 0.70489652 | 0.012147 |
| py08110.t1 | py08110.t1 | 0.488928 | 0.48341 | 0.640502 | 0.537613227 | 0.012165 |
| py05170.t1 | py05170.t1 | 0.837611 | 0.802306 | 0.759819 | 0.799912053 | 0.012398 |
| py00477.t1 | py00477.t1 | 0.864327 | 0.799309 | 0.812576 | 0.825404283 | 0.012661 |
| py01782.t1 | py01782.t1 | 0.759323 | 0.837731 | 0.776629 | 0.791227632 | 0.01273 |
| py11289.t1 | py11289.t1 | 0.755513 | 0.668615 | 0.76564 | 0.729922848 | 0.012752 |
| py06313.t1 | py06313.t1 | 0.683683 | 0.737992 | 0.787851 | 0.736508591 | 0.012783 |
| py05237.t1 | py05237.t1 | 0.826462 | 0.81976 | 0.754688 | 0.800303371 | 0.012885 |
| py05453.t1 | py05453.t1 | 0.430537 | 0.315353 | 0.542286 | 0.429392192 | 0.012927 |
| py07720.t1 | py07720.t1 | 0.804497 | 0.81097 | 0.867013 | 0.827493441 | 0.012981 |
| py02939.t1 | py02939.t1 | 0.845102 | 0.7694 | 0.815776 | 0.810092767 | 0.0132 |
| py04567.t1 | py04567.t1 | 0.775905 | 0.851269 | 0.809107 | 0.812093767 | 0.013202 |
| py01014.t1 | py01014.t1 | 0.714663 | 0.650808 | 0.57188 | 0.64578373 | 0.01332 |
| py09779.t1 | py09779.t1 | 0.604695 | 0.537124 | 0.693541 | 0.611786686 | 0.01334 |
| py03336.t1 | py03336.t1 | 0.771903 | 0.655518 | 0.706989 | 0.711469945 | 0.013347 |
| py10731.t1 | py10731.t1 | 0.673892 | 0.57389 | 0.709604 | 0.652461748 | 0.013385 |
| py10437.t1 | py10437.t1 | 0.822178 | 0.791296 | 0.73285 | 0.782107952 | 0.014145 |
| py06432.t1 | py06432.t1 | 0.619219 | 0.560143 | 0.713161 | 0.630840961 | 0.014255 |
| py10075.t1 | py10075.t1 | 0.567862 | 0.619936 | 0.718234 | 0.635344009 | 0.014304 |
| py02139.t1 | py02139.t1 | 0.760567 | 0.68366 | 0.787528 | 0.743918017 | 0.014447 |
| py00361.t1 | py00361.t1 | 0.607699 | 0.586683 | 0.726994 | 0.640458423 | 0.014448 |
| py10840.t1 | py10840.t1 | 0.657823 | 0.479948 | 0.587002 | 0.574924309 | 0.014473 |
| py11346.t1 | py11346.t1 | 0.7788 | 0.703108 | 0.799717 | 0.76054162 | 0.014688 |
| py09967.t1 | py09967.t1 | 0.626119 | 0.650509 | 0.755225 | 0.677284617 | 0.014727 |
| py07933.t1 | py07933.t1 | 0.725333 | 0.729807 | 0.817406 | 0.757515686 | 0.014938 |
| py08143.t1 | py08143.t1 | 0.664709 | 0.615781 | 0.752209 | 0.677566288 | 0.014974 |
| py11008.t1 | py11008.t1 | 0.785334 | 0.852864 | 0.845218 | 0.827805594 | 0.015027 |
| py07210.t1 | py07210.t1 | 0.765092 | 0.708782 | 0.811826 | 0.761899739 | 0.015295 |
| py04390.t1 | py04390.t1 | 0.821358 | 0.826064 | 0.748089 | 0.798503781 | 0.015336 |
| py06011.t1 | py06011.t1 | 0.778197 | 0.740125 | 0.83385 | 0.784057134 | 0.015514 |
| py10353.t1 | py10353.t1 | 0.726897 | 0.739797 | 0.822841 | 0.763178248 | 0.015735 |
| py07121.t1 | py07121.t1 | 0.813013 | 0.728831 | 0.809361 | 0.783735068 | 0.015756 |
| py01174.t1 | py01174.t1 | 0.727401 | 0.820945 | 0.79754 | 0.781961958 | 0.016212 |
| py08389.t1 | py08389.t1 | 0.705362 | 0.6792 | 0.795629 | 0.726730437 | 0.016251 |
| py07095.t1 | py07095.t1 | 0.810665 | 0.769236 | 0.85371 | 0.81120373 | 0.016279 |
| py09855.t1 | py09855.t1 | 0.749061 | 0.71567 | 0.82076 | 0.76183039 | 0.016524 |
| py04269.t1 | py04269.t1 | 0.603061 | 0.740637 | 0.706457 | 0.683384921 | 0.016637 |
| py03894.t1 | py03894.t1 | 0.857803 | 0.78284 | 0.840075 | 0.826905645 | 0.016651 |
| py07420.t1 | py07420.t1 | 0.603498 | 0.553865 | 0.719766 | 0.625709492 | 0.016818 |
| py08833.t1 | py08833.t1 | 0.747894 | 0.678861 | 0.796332 | 0.741028965 | 0.016885 |
| py01645.t1 | py01645.t1 | 0.794993 | 0.683898 | 0.686285 | 0.721725478 | 0.016899 |
| py07134.t1 | py07134.t1 | 0.844364 | 0.763514 | 0.829209 | 0.812362366 | 0.017041 |
| py05351.t1 | py05351.t1 | 0.696802 | 0.626824 | 0.76669 | 0.696771969 | 0.017272 |
| py00814.t1 | py00814.t1 | 0.757776 | 0.6607 | 0.776952 | 0.731809239 | 0.01753 |
| py03764.t1 | py03764.t1 | 0.749008 | 0.713408 | 0.610522 | 0.690979165 | 0.017579 |
| py08107.t1 | py08107.t1 | 0.658465 | 0.773941 | 0.641342 | 0.691249473 | 0.017708 |
| py09407.t1 | py09407.t1 | 0.770385 | 0.637713 | 0.727536 | 0.71187834 | 0.017915 |
| py05870.t1 | py05870.t1 | 0.807092 | 0.763856 | 0.853936 | 0.808294783 | 0.017916 |
| py09975.t1 | py09975.t1 | 0.84588 | 0.785551 | 0.861326 | 0.830919157 | 0.018186 |
| py03686.t1 | py03686.t1 | 0.761096 | 0.729792 | 0.834153 | 0.775013585 | 0.018368 |
| py03470.t1 | py03470.t1 | 0.663886 | 0.645412 | 0.779752 | 0.69634997 | 0.018635 |
| py11406.t1 | py11406.t1 | 0.799656 | 0.709936 | 0.80876 | 0.772783869 | 0.018722 |
| py06839.t1 | py06839.t1 | 0.853822 | 0.772302 | 0.766623 | 0.797582632 | 0.01882 |
| py04561.t1 | py04561.t1 | 0.829455 | 0.862198 | 0.776603 | 0.822751769 | 0.019222 |
| py04071.t1 | py04071.t1 | 0.822044 | 0.711626 | 0.725045 | 0.752904941 | 0.019248 |
| py04521.t1 | py04521.t1 | 0.809759 | 0.800285 | 0.708658 | 0.772900489 | 0.019561 |
| py00834.t1 | py00834.t1 | 0.870825 | 0.789055 | 0.799585 | 0.819821508 | 0.019718 |
| py06521.t1 | py06521.t1 | 0.680873 | 0.573354 | 0.736736 | 0.66365447 | 0.019719 |
| py03621.t1 | py03621.t1 | 0.830347 | 0.778008 | 0.864778 | 0.824377578 | 0.020014 |
| py07788.t1 | py07788.t1 | 0.783757 | 0.75918 | 0.855514 | 0.799483908 | 0.020147 |
| py08405.t1 | py08405.t1 | 0.837552 | 0.729338 | 0.755508 | 0.774132863 | 0.020199 |
| py00328.t1 | py00328.t1 | 0.850802 | 0.771972 | 0.846475 | 0.823083153 | 0.020281 |
| py07351.t1 | py07351.t1 | 0.800777 | 0.754577 | 0.854307 | 0.803220156 | 0.020777 |
| py05590.t1 | py05590.t1 | 0.842836 | 0.779927 | 0.863679 | 0.828814166 | 0.020947 |
| py04175.t1 | py04175.t1 | 0.747548 | 0.801345 | 0.850302 | 0.799731835 | 0.021256 |
| py09968.t3 | py09968.t3 | 0.766288 | 0.678081 | 0.804315 | 0.749561212 | 0.021569 |
| py10918.t1 | py10918.t1 | 0.68073 | 0.675447 | 0.804201 | 0.720126081 | 0.021853 |
| py06166.t1 | py06166.t1 | 0.736643 | 0.80226 | 0.842994 | 0.793965845 | 0.02187 |
| py06327.t1 | py06327.t1 | 0.834799 | 0.826628 | 0.740849 | 0.800758794 | 0.021996 |
| py00802.t1 | py00802.t1 | 0.662313 | 0.560409 | 0.740711 | 0.654477697 | 0.022068 |
| py07296.t1 | py07296.t1 | 0.714935 | 0.676622 | 0.812556 | 0.734704498 | 0.022485 |
| py03521.t1 | py03521.t1 | 0.811058 | 0.835734 | 0.730657 | 0.792482767 | 0.022581 |
| py05294.t1 | py05294.t1 | 0.799838 | 0.773369 | 0.869056 | 0.814087799 | 0.022744 |
| py08599.t1 | py08599.t1 | 0.722912 | 0.825135 | 0.815912 | 0.787986351 | 0.022899 |
| py00604.t1 | py00604.t1 | 0.462432 | 0.414981 | 0.66053 | 0.512647575 | 0.022991 |
| py09590.t1 | py09590.t1 | 0.800494 | 0.798821 | 0.689457 | 0.762924223 | 0.02318 |
| py02934.t1 | py02934.t1 | 0.769098 | 0.859324 | 0.840753 | 0.823058168 | 0.023326 |
| py00467.t1 | py00467.t1 | 0.740015 | 0.701177 | 0.829111 | 0.756767474 | 0.023393 |
| py08509.t1 | py08509.t1 | 0.796639 | 0.792087 | 0.8774 | 0.822042061 | 0.023399 |
| py04464.t1 | py04464.t1 | 0.697356 | 0.666561 | 0.808169 | 0.724028714 | 0.023427 |
| py01689.t1 | py01689.t1 | 0.787124 | 0.878157 | 0.810661 | 0.825314168 | 0.023532 |
| py05886.t1 | py05886.t1 | 0.834916 | 0.709547 | 0.759984 | 0.768149287 | 0.023799 |
| py09391.t1 | py09391.t1 | 0.804319 | 0.678868 | 0.781261 | 0.754815967 | 0.023844 |
| py04192.t1 | py04192.t1 | 0.730081 | 0.834832 | 0.817573 | 0.794162157 | 0.023929 |
| py00411.t1 | py00411.t1 | 0.846253 | 0.729991 | 0.790681 | 0.788975026 | 0.024389 |
| py05784.t1 | py05784.t1 | 0.864013 | 0.766714 | 0.830899 | 0.82054177 | 0.024406 |
| py07223.t1 | py07223.t1 | 0.872954 | 0.774648 | 0.799509 | 0.815703458 | 0.024695 |
| py00619.t1 | py00619.t1 | 0.808654 | 0.729987 | 0.842038 | 0.793559627 | 0.024923 |
| py00656.t1 | py00656.t1 | 0.875148 | 0.78172 | 0.841691 | 0.832852995 | 0.025709 |
| py10200.t1 | py10200.t1 | 0.75023 | 0.598266 | 0.743979 | 0.697491859 | 0.025892 |
| py07876.t1 | py07876.t1 | 0.779831 | 0.775238 | 0.87167 | 0.808913349 | 0.025966 |
| py01362.t1 | py01362.t1 | 0.87184 | 0.820647 | 0.769759 | 0.820748964 | 0.025978 |
| py08618.t1 | py08618.t1 | 0.792971 | 0.799185 | 0.882415 | 0.824856985 | 0.026051 |
| py06248.t1 | py06248.t1 | 0.52194 | 0.486941 | 0.713067 | 0.573982565 | 0.026147 |
| py00151.t1 | py00151.t1 | 0.788807 | 0.744271 | 0.859111 | 0.797396236 | 0.02616 |
| py05131.t1 | py05131.t1 | 0.849583 | 0.752178 | 0.838961 | 0.813573981 | 0.026309 |
| py04680.t1 | py04680.t1 | 0.795783 | 0.709049 | 0.62791 | 0.710913952 | 0.026979 |
| py09341.t1 | py09341.t1 | 0.779077 | 0.829915 | 0.696152 | 0.768381158 | 0.027177 |
| py08216.t1 | py08216.t1 | 0.7386 | 0.775877 | 0.858483 | 0.79098682 | 0.02754 |
| py11433.t1 | py11433.t1 | 0.836908 | 0.832934 | 0.733788 | 0.801210257 | 0.027605 |
| py08787.t1 | py08787.t1 | 0.890079 | 0.801554 | 0.807098 | 0.83291036 | 0.028125 |
| py07492.t1 | py07492.t1 | 0.805322 | 0.712002 | 0.835939 | 0.784421326 | 0.028616 |
| py10518.t1 | py10518.t1 | 0.812822 | 0.684138 | 0.799957 | 0.765639163 | 0.029158 |
| py03465.t1 | py03465.t1 | 0.825416 | 0.784386 | 0.886493 | 0.83209803 | 0.029825 |
| py10051.t1 | py10051.t1 | 0.85489 | 0.773247 | 0.868139 | 0.832092111 | 0.029834 |
| py11134.t1 | py11134.t1 | 0.78807 | 0.827859 | 0.689652 | 0.768527147 | 0.030077 |
| py06172.t1 | py06172.t1 | 0.738508 | 0.763775 | 0.861918 | 0.788067065 | 0.030124 |
| py09890.t1 | py09890.t1 | 0.705609 | 0.771664 | 0.84648 | 0.774584362 | 0.031077 |
| py00548.t1 | py00548.t1 | 0.595597 | 0.598936 | 0.782024 | 0.658852481 | 0.031085 |
| py06124.t1 | py06124.t1 | 0.557521 | 0.565051 | 0.765258 | 0.629276527 | 0.03206 |
| py06711.t1 | py06711.t1 | 0.842074 | 0.751556 | 0.860124 | 0.817917623 | 0.032383 |
| py06094.t1 | py06094.t1 | 0.806005 | 0.887162 | 0.779062 | 0.824076533 | 0.032452 |
| py02930.t1 | py02930.t1 | 0.878199 | 0.790415 | 0.761543 | 0.810052024 | 0.032452 |
| py08997.t1 | py08997.t1 | 0.553761 | 0.767045 | 0.685901 | 0.668902142 | 0.033479 |
| py11283.t1 | py11283.t1 | 0.837381 | 0.825907 | 0.715037 | 0.792775142 | 0.033659 |
| py05607.t1 | py05607.t1 | 0.704627 | 0.66588 | 0.831851 | 0.734119192 | 0.033758 |
| py00602.t1 | py00602.t1 | 0.818904 | 0.766533 | 0.883241 | 0.822892725 | 0.034448 |
| py07129.t1 | py07129.t1 | 0.876331 | 0.768409 | 0.75475 | 0.799829871 | 0.034979 |
| py08028.t1 | py08028.t1 | 0.837175 | 0.764661 | 0.879181 | 0.827005639 | 0.035409 |
| py03225.t1 | py03225.t1 | 0.815892 | 0.641949 | 0.640828 | 0.699556418 | 0.035501 |
| py02671.t1 | py02671.t1 | 0.813971 | 0.676482 | 0.810019 | 0.766823873 | 0.035561 |
| py04662.t1 | py04662.t1 | 0.774483 | 0.835077 | 0.887172 | 0.832243876 | 0.035671 |
| py03998.t1 | py03998.t1 | 0.81063 | 0.779173 | 0.891852 | 0.827218346 | 0.035734 |
| py05032.t1 | py05032.t1 | 0.735365 | 0.717211 | 0.860313 | 0.770963113 | 0.036473 |
| py07757.t1 | py07757.t1 | 0.774049 | 0.830212 | 0.88916 | 0.831140439 | 0.03662 |
| py09652.t1 | py09652.t1 | 0.708934 | 0.840318 | 0.823676 | 0.790975959 | 0.036895 |
| py08776.t1 | py08776.t1 | 0.838662 | 0.722778 | 0.842668 | 0.801369418 | 0.037011 |
| py00767.t1 | py00767.t1 | 0.662764 | 0.819284 | 0.790916 | 0.757654733 | 0.037277 |
| py05543.t1 | py05543.t1 | 0.763742 | 0.833909 | 0.88318 | 0.82694379 | 0.037838 |
| py01907.t1 | py01907.t1 | 0.694036 | 0.615714 | 0.816389 | 0.708713054 | 0.037915 |
| py04935.t1 | py04935.t1 | 0.675428 | 0.600945 | 0.811017 | 0.69579688 | 0.038516 |
| py10502.t1 | py10502.t1 | 0.780177 | 0.742501 | 0.87817 | 0.800282886 | 0.038629 |
| py01142.t1 | py01142.t1 | 0.803743 | 0.730343 | 0.869684 | 0.801256614 | 0.038641 |
| py02330.t1 | py02330.t1 | 0.732645 | 0.800685 | 0.871933 | 0.801754283 | 0.038768 |
| py08108.t1 | py08108.t1 | 0.782226 | 0.74788 | 0.880883 | 0.803662801 | 0.038836 |
| py01681.t1 | py01681.t1 | 0.595734 | 0.72089 | 0.801158 | 0.705927302 | 0.038916 |
| py03051.t1 | py03051.t1 | 0.664765 | 0.836187 | 0.772984 | 0.757978432 | 0.040206 |
| py02966.t1 | py02966.t1 | 0.832354 | 0.713494 | 0.845497 | 0.797114975 | 0.040251 |
| py02267.t1 | py02267.t1 | 0.844339 | 0.718268 | 0.664261 | 0.742289221 | 0.040288 |
| py07445.t1 | py07445.t1 | 0.656627 | 0.505757 | 0.763764 | 0.642049443 | 0.041038 |
| py03568.t1 | py03568.t1 | 0.740281 | 0.80587 | 0.879939 | 0.80869646 | 0.041705 |
| py08743.t1 | py08743.t1 | 0.89683 | 0.813031 | 0.771527 | 0.827129077 | 0.042564 |
| py08533.t1 | py08533.t1 | 0.642825 | 0.81365 | 0.792107 | 0.749527088 | 0.043039 |
| py07908.t1 | py07908.t1 | 0.834372 | 0.752531 | 0.883485 | 0.823462943 | 0.04376 |
| py00220.t1 | py00220.t1 | 0.875057 | 0.836547 | 0.741809 | 0.817804515 | 0.044117 |
| py08069.t1 | py08069.t1 | 0.785799 | 0.760992 | 0.893728 | 0.813506232 | 0.044566 |
| py00532.t1 | py00532.t1 | 0.63499 | 0.798898 | 0.804013 | 0.745966839 | 0.044577 |
| py08713.t1 | py08713.t1 | 0.892282 | 0.75513 | 0.813852 | 0.820421468 | 0.04562 |
| py04302.t1 | py04302.t1 | 0.759601 | 0.749555 | 0.888097 | 0.79908432 | 0.045912 |
| py04817.t1 | py04817.t1 | 0.799895 | 0.78899 | 0.906301 | 0.831728535 | 0.04606 |
| py08528.t1 | py08528.t1 | 0.841528 | 0.736671 | 0.872 | 0.816733059 | 0.046551 |
| py11133.t1 | py11133.t1 | 0.568547 | 0.540927 | 0.798485 | 0.635986338 | 0.046797 |
| py00652.t1 | py00652.t1 | 0.810809 | 0.89791 | 0.760814 | 0.823177742 | 0.047678 |
| py06804.t1 | py06804.t1 | 0.729038 | 0.751973 | 0.883317 | 0.788109142 | 0.047791 |
| py03411.t1 | py03411.t1 | 0.824652 | 0.846837 | 0.691804 | 0.787764174 | 0.048282 |
| py11116.t1 | py11116.t1 | 0.68643 | 0.590898 | 0.826333 | 0.701220139 | 0.048573 |
| py06491.t1 | py06491.t1 | 0.741114 | 0.840567 | 0.879191 | 0.820290624 | 0.048591 |
| py08113.t1 | py08113.t1 | 0.84025 | 0.728741 | 0.869795 | 0.812928774 | 0.048878 |
| py07624.t1 | py07624.t1 | 0.723289 | 0.684267 | 0.867074 | 0.758209956 | 0.048998 |
| py06621.t1 | py06621.t1 | 0.90104 | 0.80514 | 0.763362 | 0.823180742 | 0.049235 |
| py09952.t1 | py09952.t1 | 0.749224 | 0.81456 | 0.893658 | 0.819147529 | 0.049395 |
| py05486.t1 | py05486.t1 | 0.587745 | 0.744765 | 0.810292 | 0.714267215 | 0.049471 |
